# Analyzing the Behavior of Neuronal Pathways in Alzheimer's Disease Using Petri Net Modeling Approach

**DOI:** 10.3389/fninf.2018.00026

**Published:** 2018-05-23

**Authors:** Javaria Ashraf, Jamil Ahmad, Amjad Ali, Zaheer Ul-Haq

**Affiliations:** ^1^Research Center for Modeling and Simulation, National University of Sciences and Technology, Islamabad, Pakistan; ^2^Atta-Ur-Rahman School of Applied Biosciences, National University of Sciences and Technology, Islamabad, Pakistan; ^3^Dr. Panjwani Center for Molecular Medicine and Drug Research, International Center for Chemical Sciences, University of Karachi, Karachi, Pakistan

**Keywords:** Calpain, CAST, calcium, PKC, APP, Stochastic petri net, Alzheimer disease, Amyloid beta

## Abstract

Alzheimer's Disease (AD) is the most common neuro-degenerative disorder in the elderly that leads to dementia. The hallmark of AD is senile lesions made by abnormal aggregation of amyloid beta in extracellular space of brain. One of the challenges in AD treatment is to better understand the mechanism of action of key proteins and their related pathways involved in neuronal cell death in order to identify adequate therapeutic targets. This study focuses on the phenomenon of aggregation of amyloid beta into plaques by considering the signal transduction pathways of Calpain-Calpastatin (CAST) regulation system and Amyloid Precursor Protein (APP) processing pathways along with Ca^2+^ channels. These pathways are modeled and analyzed individually as well as collectively through Stochastic Petri Nets for comprehensive analysis and thorough understating of AD. The model predicts that the deregulation of Calpain activity, disruption of Calcium homeostasis, inhibition of CAST and elevation of abnormal APP processing are key cytotoxic events resulting in an early AD onset and progression. Interestingly, the model also reveals that plaques accumulation start early (at the age of 40) in life but symptoms appear late. These results suggest that the process of neuro-degeneration can be slowed down or paused by slowing down the degradation rate of Calpain-CAST Complex. In the light of this study, the suggestive therapeutic strategy might be the prevention of the degradation of Calpain-CAST complexes and the inhibition of Calpain for the treatment of neurodegenerative diseases such as AD.

## 1. Introduction

**Figure 1:**
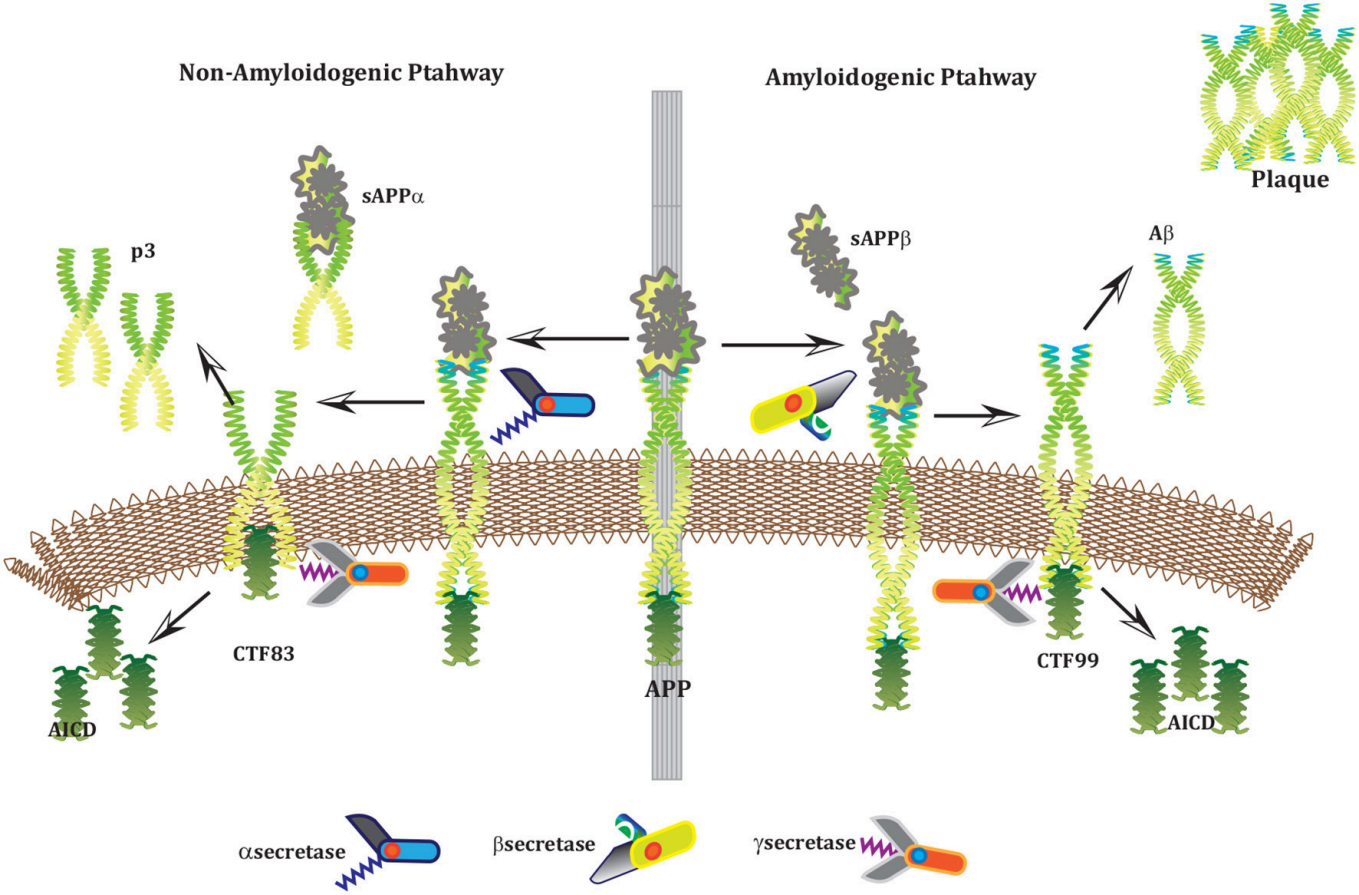
APP and processing products: APP is synthesized in the ER and then transported to the trans-Golgi-network (TGN) where it is cleaved by secretases. In non-amyloidogenic pathway (**left**), cleavage of APP by α-secretase results in the generation of sAPPα and C-terminal fragments CTF83 which is further cleaved by γ-secretase into p3 and AICD. Proteolysis by α-secretase prevents *Aβ* production as the cleavage site in APP is within the *Aβ* domain. In amyloidogenic pathway (**right**), APP is cleaved into sAPPβ and CTF99 by β−secretase / BACE activity. Furthermore, CTF99 breaks down into AICD and *Aβ* by γ-secretase activity. *Aβ* fragments oligomerize and fibrillize into plaques.

Alzheimer's disease (AD) is a neurodegenerative disorder which has impacted nearly 44 million^1^ people around the world and this number is still increasing. AD is the leading cause of dementia in the old age (Ashford, 2004). Unfortunately, it is diagnosed only in one out of four people living with the disease^1^. Clinical characterization of AD includes memory loss and cognitive impairment which further lead to damaged behavioral activities and render a person completely dependent on an external aid (Budson and Price, 2005). AD establishes over time with the appearance of pathological emblems which are senile plaques and neurofibrillary tangles. These lesions comprise of extracellular deposits of Amyloid beta (*Aβ*) (Selkoe, 2000; Golde, 2005; Tam and Pasternak, 2012) and intracellular self-gathered clumps of tau proteins (Lee et al., 2001), respectively. *Aβ* is a 40–42 amino-acids long peptide which is formed after the proteolytic cleavage of Amyloid Precursor Protein (APP) (Selkoe, 2000; Golde, 2005; Tam and Pasternak, 2012). Previous studies have shown that *Aβ* monomers are initially non-toxic but their conversion to oligomers makes them toxic (Volles and Lansbury, 2002; Walsh and Selkoe, 2004). Eventually, the abnormal accumulation of oligomers form plaques (Walsh et al., 2002) that deposit into neuronal Endoplasmic Reticulum (ER) (Cuello, 2005) and in extracellular space (Trojanowski and Lee, 2000; Walsh et al., 2000). Aggregation of senile plaques and neurofibrillary tangles cause neuronal cell death and synaptic failure (Tiraboschi et al., 2000; Selkoe, 2002). During the last two decades, several lines of studies have pointed toward the imbalance between *Aβ* production and its clearance plays a central role in pathogenesis of AD. Since 1992, this hypothesis has earned acquiescence (Hardy and Higgins, 1992) and is known as “***Amyloid cascade hypothesis (ACH)***”. It suggests that *Aβ* and processing of APP are crucial in neuro-degeneration. In AD, aggregation of *Aβ* is the first step leading toward the formation of senile plaques (Hardy and Selkoe, 2002; Vassar, 2005). APP is a type1 trans-membrane protein produced in ER (Greenfield et al., 1999; Roussel et al., 2013). In neurons, production and metabolism of APP occurs rapidly which makes it a crucial element in neuro-pathogenesis (Lee et al., 2008). The main APP proteolytic processing steps occur at the cell surface and Trans-Golgi networks (TGNs). Proteolysis of APP can occur through the so-called *non-amyloidogenic* and *amyloidogenic Pathways* (Figure 1). The first step of non-amyloidogenic pathway is carried out by the enzyme alpha (α)-secretase that breaks down APP into soluble Amyloid precursor protein alpha (sAPPα) and alpha C-terminal fragment (αCTF / CTF83). The catalysis by α-secretase is imperative as it cuts APP within *Aβ* domain which blocks *Aβ* formation (Lichtenthaler, 2011). This initial step can also be driven by the beta (β)-secretase / β-site APP-cleaving enzyme (BACE), a transmembrane aspartyl protease (Vassar et al., 1999; Haass, 2004) (Figure 1), which constitute amyloidogenic pathway. BACE is a crucial enzyme, that acts as a rate limiting protein in *Aβ* generation. It breaks down the APP into soluble Amyloid precursor protein beta (sAPPβ) and beta C-terminal fragments (βCTF / CTF99) (Cai et al., 2001). The CTFs are intermediate products of the first step in both pathways which remain attached to the membrane and they are further cleaved by gamma (γ)-secretase (Zhang et al., 2011). In non-amyliodogenic pathway, the fragment α-CTF is cut down by γ-secretase into p38 and the Amyloid Precursor Protein Intracellular Cytoplasmic / C-terminal Domain (AICD). While in amyloidogenic pathway, γ-secretase degrades the βCTF into *Aβ* and AICD (O'Brien and Wong, 2011) (Figure 1).

**Figure 2:**
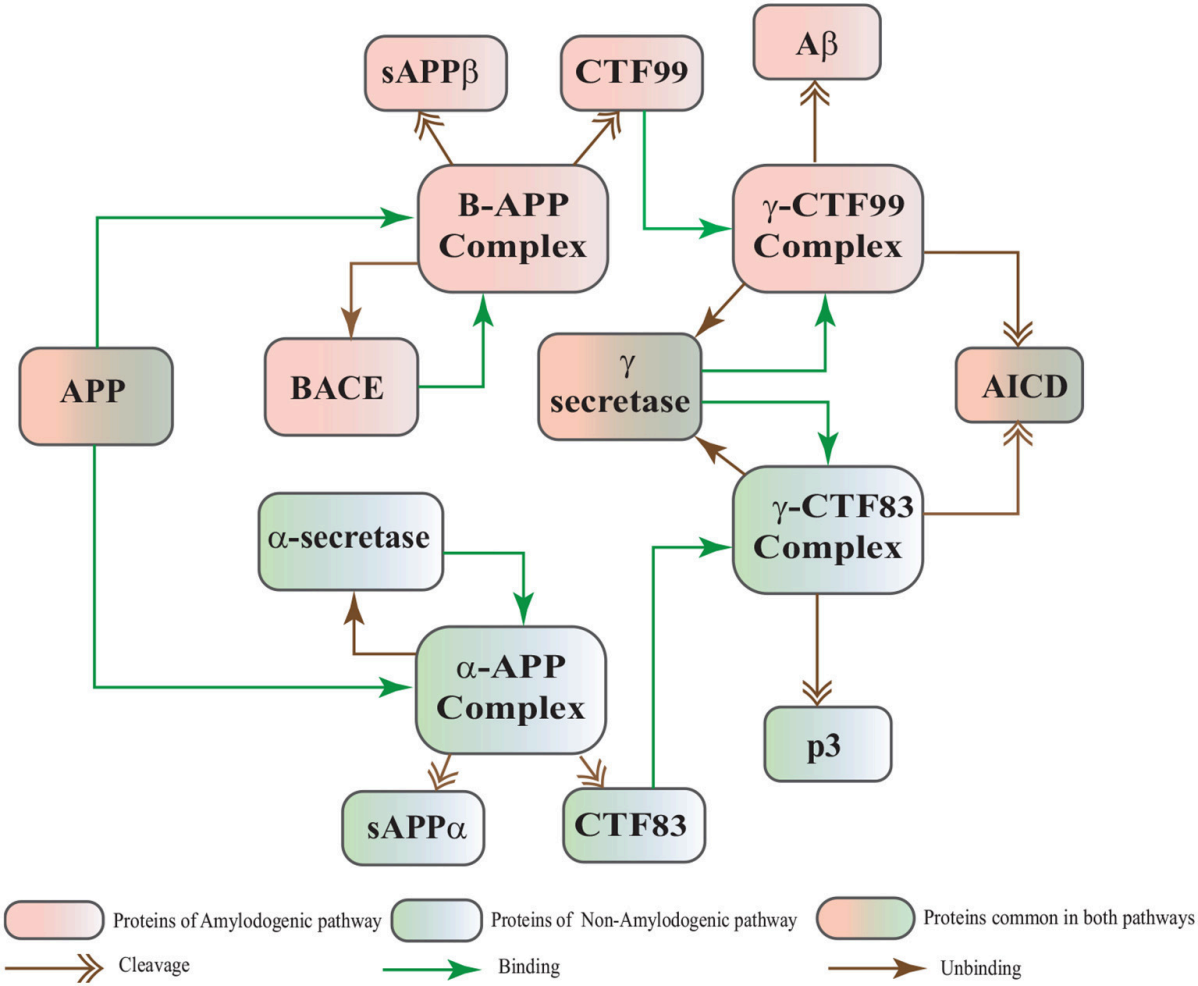
APP processing pathways BRN derived from Figure 1.

The Biological Regulatory Networks (BRN) of APP processing, depicted in Figure 2, is also built from Figure 1. APP processing depends on sequential cleavage by three secretases (α/β-secretase and γ-secretase). In normal conditions, α-secretase residing at the plasma membrane is constitutively active for APP coming to the cell surface and thus favoring non-amyloidogenic pathway (De Strooper and Annaert, 2000). Though there is an interesting fact about APP proteolysis that none of the secretases show special substrate specificity toward APP. There are several transmembrane proteins such as cell surface receptors and ligand, growth factors and cytokines besides APP which undergo ectodomain shedding by enzymes with α-secretase activity (Annaert and Saftig, 2009). In the same manner, BACE shows low affinity toward APP and it is not its exclusive physiological substrate (DeStrooper et al., 2006; Hu et al., 2006). Many observations highlight that in healthy cells APP is frequently processed through non-amyloidogenic pathway to resist amyloid generation while it is altered in pathological conditions (De Strooper and Annaert, 2000). Abnormal processing of APP is stated to be the first and fundamental step in plaques formation in AD pathogenesis (Jonsson et al., 2012). In neuropathological conditions, BACE affinity toward APP increases two folds which leads to enhanced *Aβ* production (Yang et al., 2003; Li and Südhof, 2004). Recent studies on transgenic mice model have shown that BACE activity is modulated by Calpain activation in AD pathology (Liang et al., 2010). Calpain-Calpastatin system also plays a key role in neurodegeneration. Transgenic mice models have shown that over expression of APP, increased production of *Aβ*, inhibition of Calpastatin (CAST) and activation of Calpain increase neuronal degeneration in AD (Higuchi et al., 2012).

Calpains are protein clan of cysteine/ thiol proteases and their activity depends on Ca^2+^ concentration (Ferreira, 2012). The most studied Calpains, mu(μ)-Calpain (Calpain1) and m-Calpain (Calpain2) are present abundantly in neurons, central nervous system (CNS) and glial cells. Though their distribution differs, Calpain1 is ubiquitous and expressed more in neurons while Calpain2 is present in glial cells (Ono and Sorimachi, 2012; Santos et al., 2012). Calpain1 requires micro-molar concentration of Ca^2+^ (10–50μM), while Calpain2 is activated by mili-molar concentration of Ca^2+^ (250–350μM) *in vitro* (Goll et al., 2003; Ryu and Nakazawa, 2014). Ca^2+^ plays important role in ensuring the cell's vital functions. In addition to calcium, Calpain is tightly regulated in the cell by CAST which is also ubiquitous and solely a specific endogenous inhibitor for both Calpains (Melloni et al., 2006).

**Figure 3:**
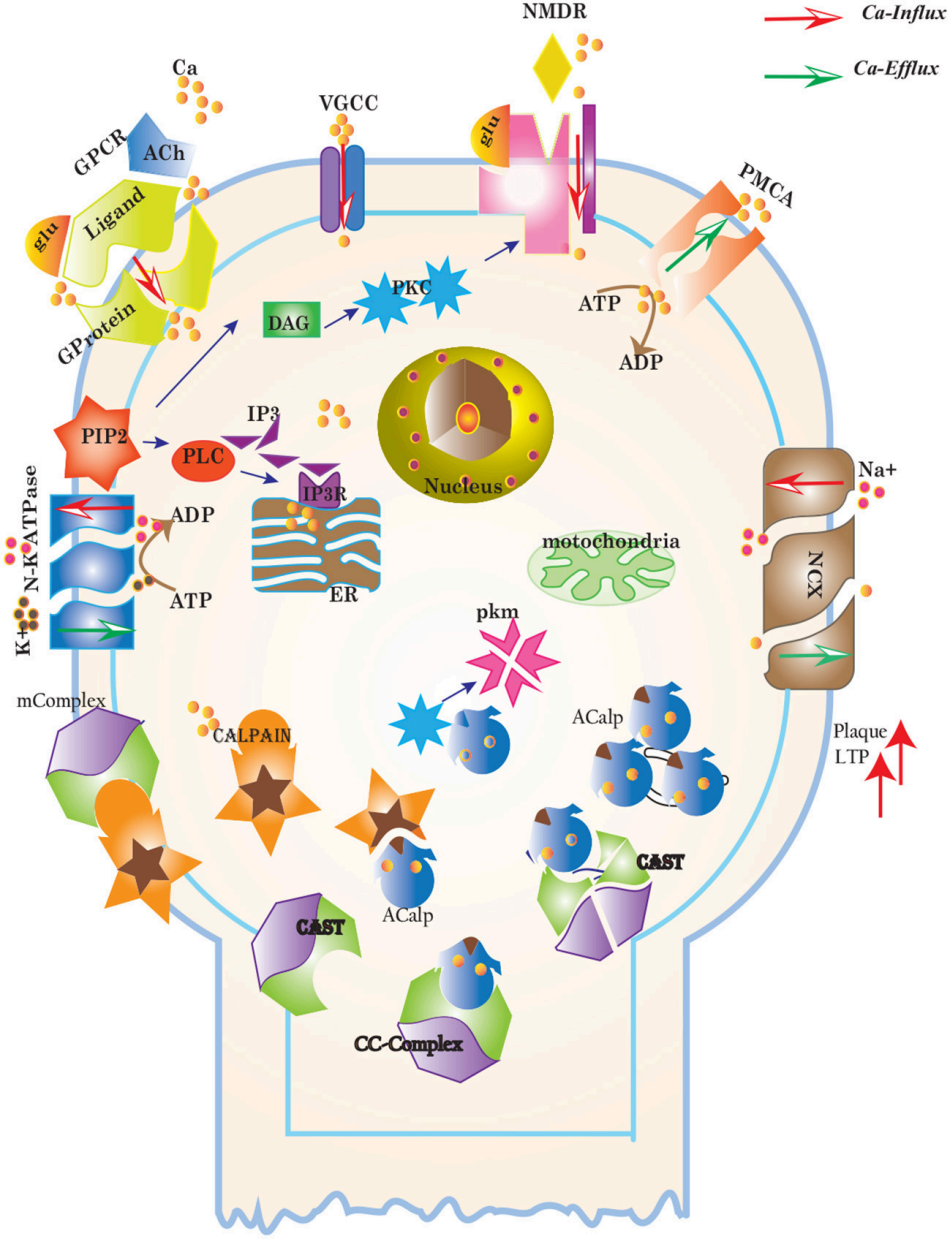
Different calcium channels work in harmony to establish homeostasis in neurons. Calcium influx is controlled by voltage gated (*VGCCs*) or receptor-ligand based (*NMDAR, GPCR*) channels. *ER* also release *Ca*^2+^ into the cytoplasm through inositol-1,4,5-trisphosphate (*IP3R*) and ryanodine receptors. Calcium efflux is carried out by energy (*ATP*) dependent channels such as plasma membrane calcium ATPase (*PMCA*), sodium-potassium ATPase (*NKA*) and sodium-calcium exchanger (*NCX*) channels. Calcium homeostasis influences Calpain-CAST system. At membrane, Calpain is bound to CAST to form *mComplex* at low *Ca*^2+^ level. At high *Ca*^2+^ concentration, Calpain is released into cytoplasm and autolysed to active form *ACalp* that again forms complex with CAST (*cComplex*). Gradually the complex breaks down and releases *ACalp* which enhances *Plaque* accumulation and *LTP* events.

CAST is reported as an explicit suicide substrate for Calpain (Yang et al., 2013). The proportion of CAST in a cell is normally larger than Calpain, its ratio with location is crucial in controlling the extent of activation of Calpain within a cell (Todd et al., 2003). CAST interacts with Calpain at different stages i.e., first it constrains Calpain at the membrane where pro-Calpain is attached then it interacts with active Calpain inside cytosol (Hanna et al., 2008). CAST forms a reversible complex with Calpain at both the sites. At membrane, the reversible complex breaks down when Ca^2+^ influx increases to release Calpain. Inside cytosol, Calpain undergoes autolysis to attain active conformation. In response, CAST changes its cellular distribution to make itself widely available in the cytoplasm to counter active Calpain (Todd et al., 2003). Both active Calpain and CAST rejoin in a reversible complex to resist persistent activity of Calpain (De Tullio et al., 1999). Active Calpain modulates CAST by slowly digesting it into small inactive fragments which results in plethora of Calpain in cell leading to pathological condition (Averna et al., 2001b; Tompa et al., 2002) (Figure 3). It has been reported that in AD CAST becomes depleted from different regions of the brain as compared to healthy aged brain (Rao et al., 2008). It has also been observed that by controlling Calpain, CAST is indirectly preventing cell membrane damages induced by high Ca^2+^ and *Aβ* peptide (Vaisid et al., 2008).

**Figure 4:**
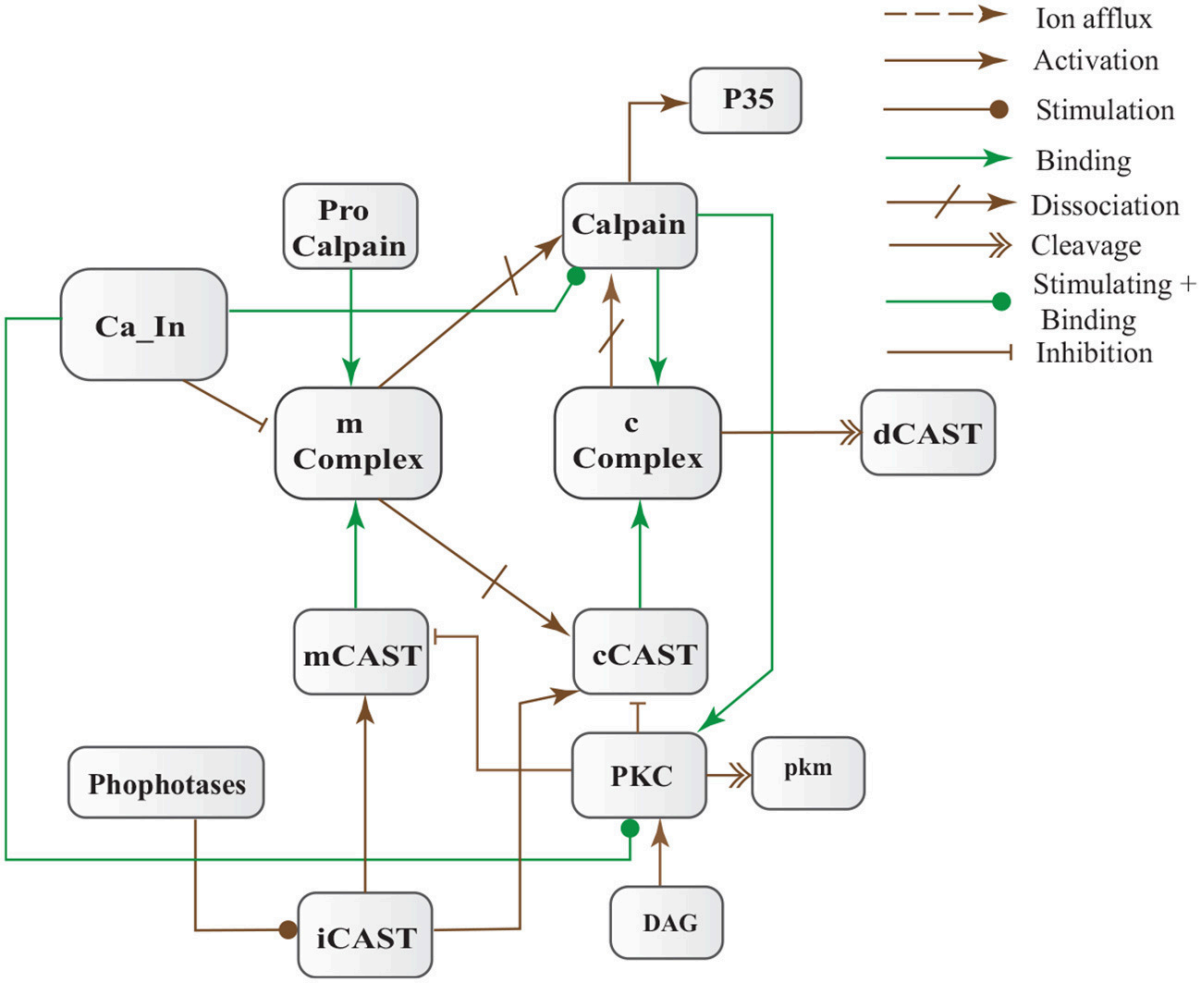
Calpain-CAST system BRN derived from Figure 3.

CAST pool is regulated by reversible phosphorylation via PKC, which is a Ca^2+^-activated phospholipid dependent kinase. Moreover, it is de-phosphorylated by protein phosphatases (ppase) (Melloni et al., 2006). Phosphorylation control CAST inhibitory efficiency in brain (Averna et al., 2001a) to regulate its availability for calpain inhibition. Reversible protein phosphorylation regulates many neuronal functions and is important for neuronal signal transduction (Wu and Lynch, 2006). Inactive PKC is converted to Ca^2+^-bound activated form in the presence of diacylglycerol (DAG) which in turn is activated by receptor based hydrolysis of phosphoinositides 3 (IP3) (Courjaret et al., 2003). The N-terminal region of CAST which is responsible for the function of the protein has a site for phosphorylation by PKC. CAST is phosphorylated by PKC to decrease its inhibitory efficiency toward calpain (Averna et al., 2001a) (Figure 3). It has been observed that PKC also regulates APP processing by activating α-secretase (Rossner et al., 2001; Racchi et al., 2003), it promotes non-amylodogenic pathway over β-secretase (Lanni et al., 2004). *In vivo* studies show that in the presence of PKC, secretion of sAPPα increases and *Aβ* secretion declines (Chen and Fernandez, 2004). Other studies about AD found that PKC has substantial role in AD pathology (Etcheberrigaray et al., 2004; Alkon et al., 2007). Active Calpain also interacts with PKC and converts it into constitutive active enzyme (Yamakawa et al., 2001; Goll et al., 2003). Calpain1 directly starts depletion of PKC from cell by converting it into protein kinase M (PKM) (Yamakawa et al., 2001; Liu et al., 2008). The whole mechanism is also depicted in the form of Calpain-CAST system BRN in Figure 4.

**Figure 5:**
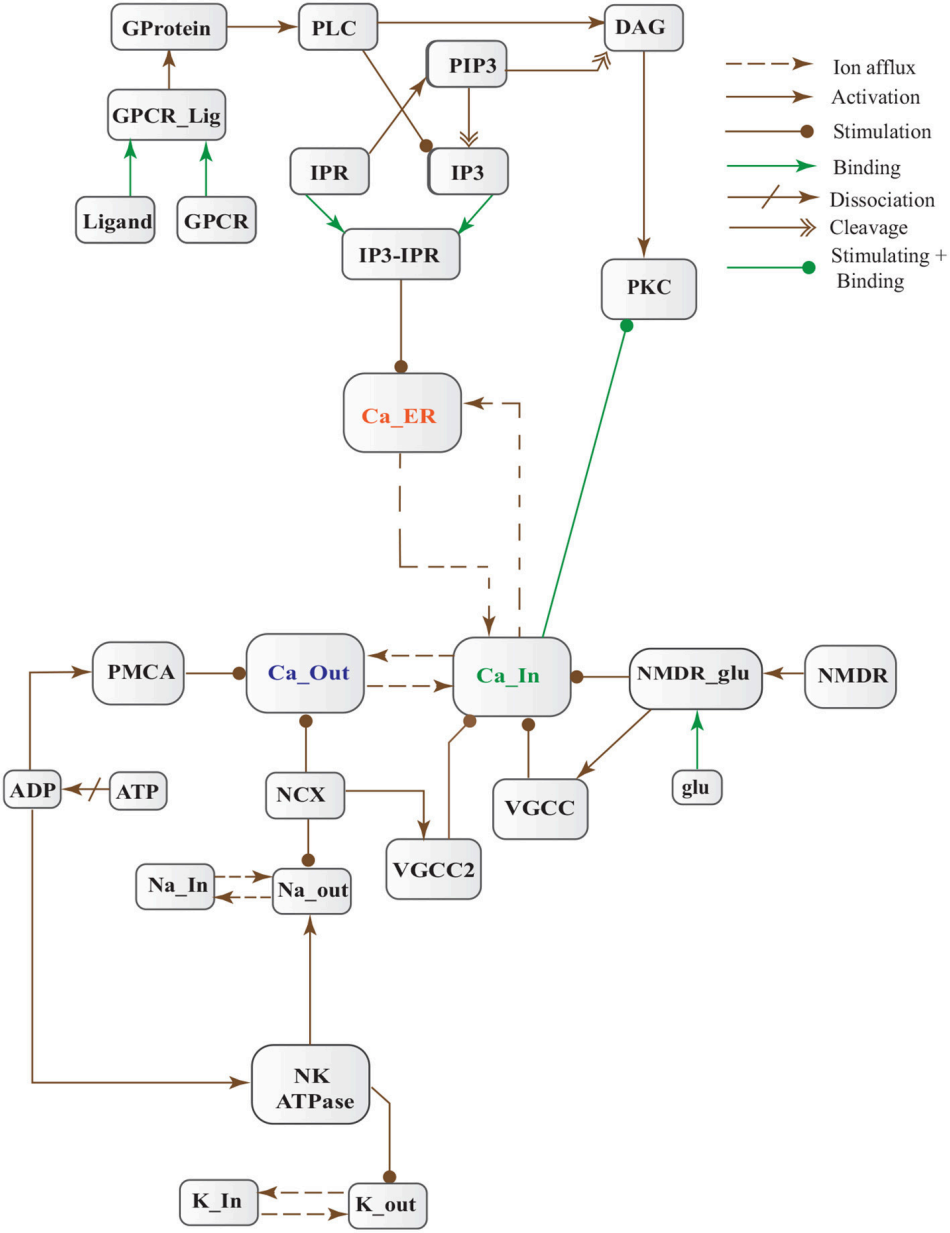
Calcium Influx Efflux BRN derived from Figure 3.

The dysregulation of Calcium homeostasis contributes in aging and neurodegeneration (Mattson, 2004; Smith et al., 2005; Stutzmann, 2005). A tremendous deal of work by calcium is tightly regulated in time, space and intensity by intracellular stores, influx and efflux channels (Stutzmann, 2005). At resting stage, extracellular Ca^2+^ concentration ranges from 1.5 to 2.0 mM (Orrenius et al., 2003). While magnitude of Ca^2+^ inside a cell is very low (between 50–100/ 50–300 nM) (LaFerla, 2002; Orrenius et al., 2003) and after activation it can rise to several micromoles. On contrary, inside ER, the level of Ca^2+^ is in the range 100-500μM (LaFerla, 2002) which is approximately 1000 times higher than cytosol concentration at the resting phase. Persistent alteration of Ca^2+^ homeostasis affects production and digestion of pathological proteins such as Calpain, *Aβ* and tau protein. Dysregulation of cellular Ca^2+^ level is an early and main feature of AD (Mattson et al., 2000; LaFerla, 2002; Small, 2009).

Cytosolic Ca^2+^ is maintained at very low level as compared to extracellular space through several homeostatic mechanisms, working both temporally and spatially (Figure 3). These equilibrating apparatuses include voltage-operated channels (VOCs) and receptor operated channels (ROCs) for Ca^2+^ inclusion, Ca^2+^ storage in organelles e.g., ER (Wojda et al., 2008) and Ca^2+^ extrusion to extracellular space. Different ATP-dependent membrane pumps such as plasma membrane calcium ATPase channel (PMCA) and sodium-calcium exchanger (NCX) which are dependent on sodium-potassium ATPase (NKA) (Wojda et al., 2008; Brittain et al., 2012) are used for Ca^2+^ efflux. In different physiological processes, elevation of Ca^2+^ is necessary to switch-on respective proteins. Ca^2+^ inclusion is administered by several routes such as N-methyl-D-aspartate receptor (NMDAR), an imperative type of ROCs, which switch into open conformation after binding of endogenous glutamate (glu) as ligand. Another important influx gateway is voltage gated Ca^2+^ channel (VGCC) which is in closed conformation when neuronal membrane is polarized (Schmolesky et al., 2002; Cain and Snutch, 2011). The VGCC adopts open conformation as plasma membrane depolarizes due to Ca^2+^/ sodium (Na+) influx through ROCs or ion channels (Weber, 2012). Ca^2+^ influx also increases from intracellular stores in ER through store-operated channels. There are two calcium channels in ER which are IP3-sensitive and ryanodine (RyRs)-sensitive Ca^2+^ stores (Berridge, 2009). IP3 driven release of Ca^2+^ starts by binding of G-protein coupled receptor (GPCR) on plasma membrane which induces Phospholipase C (PLC) mediated cleavage of phosphatidylinositol-4,5-bisphosphate (PIP2) on cell membrane into DAG and IP3. IP3 binds to its receptor on ER membrane and stimulate Ca^2+^ release into the cytoplasm (Berridge, 2009; Krebs et al., 2015). Furthermore, depletion of ER stores mediate influx of extracellular Ca^2+^ through store-operated channels (SOCs) (Emptage et al., 2001; Weber, 2012). The mechanism for lowering Ca^2+^ from cell is controlled by PMCA and NCX. Both PMCA and NCX are energy dependent while, NCX is also Na^+^ gradient dependent (Wojda et al., 2008). The BRN of Calcium channels, Figure 4, is also helpful in understanding the mechanism underlying the Ca^2+^ homeostasis.

To comprehend the above mentioned neuronal pathways, models are constructed to understand their dynamics. Stochastic approaches describe the randomness of biological system accurately as compared to ordinary differential equations. In BRNs, the activation or inhibition processes take place with random time delays, therefore, stochastic modeling frameworks are more suitable for their modeling. Petri nets provide complementary approach for both qualitative and quantitative modeling and simulation of the dynamical behavior of large systems in an intuitive way (Mounts and Liebman, 1997; Tsavachidou and Liebman, 2002; Tareen and Ahmad, 2015). The study (Tsavachidou and Liebman, 2002) shows that the Petri net models predict the experimental findings which support the soundness of these models. Stochastic petri nets (SPNs) have emerged as a promising tool for modeling and analyzing BRNs in the field of molecular biology (Goss and Peccoud, 1998). The dynamic behaviors of a variety of BRNs have been studied using stochastic simulations (Mura and Csikász-Nagy, 2008; Lamprecht et al., 2011; Castaldi et al., 2012; Marwan et al., 2012).

**Figure 6:**
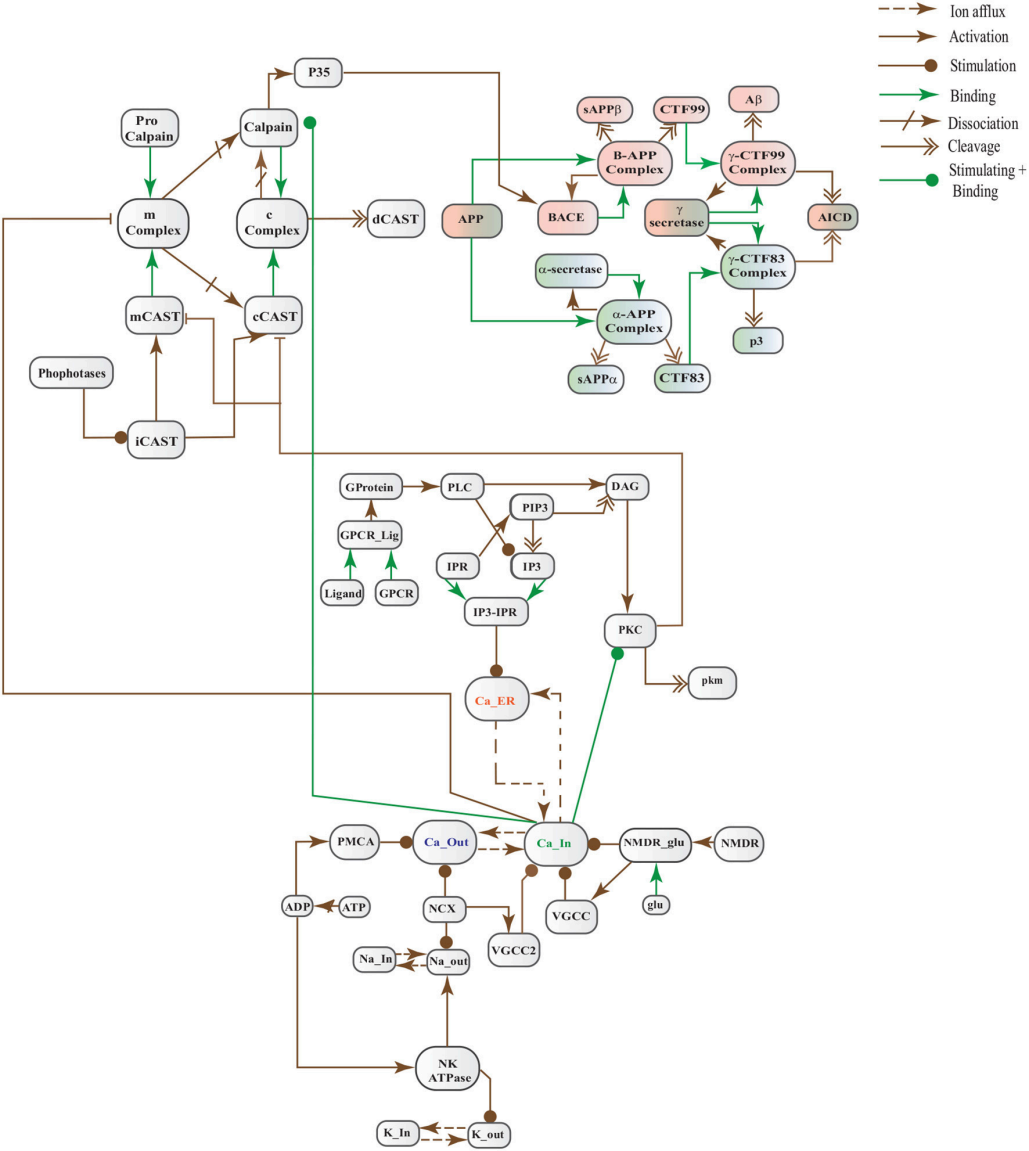
A crosstalk network of three pathways: APP processing pathways, Calcium Channels and Calpain-CAST system.

In this study, we have modeled and analyzed the neuronal physiological system constituting Ca^2+^ channels maintaining homeostasis, CAST regulating Calpain system and APP processing pathways separately and collectively at molecular level using SPNs to understand the AD progression mechanism. Particularly, we have analyzed neuronal patho-physiological dynamic behaviors causing the development of hallmark lesions in brain to answer many question e.g., how dysregulation of Ca^2+^ triggers AD? When CAST, the sole inhibitor of Calpain, depletes from the brain cells? how *Aβ* production increases? and when the accumulation of plaques start? The answers to these questions lie in the modeling of the combined BRN Figure 6. The model predicts that Calpain is the main cause of dysregulation, which start with the rise in Ca^2+^ levels in the cytosol. Calpain activates different pathways through which *Aβ* production and accumulation increases. Plaques start building with time at the age of forty and older. Plaques first enter lag phase and then into rapid growth phase. Calpain slowly degrades CAST which depletes from the cell and eventually neuronal degradation progresses. These results suggest that patho-physiological events such as dysregulation of Ca^2+^ homeostasis, Calpain hyper-activation, CAST degradation and abnormal digestion of APP, all are inter-connected and a cumulative study of these processes through SPN was needed.

## 2. Methodology

Petri nets (PNs) have three components namely places, transitions and edges. Place and transition are collectively known as nodes/ vertices of a PN. An arc or edge joins nodes such as a place (*pre-place*) to a transition or a transition to a place (*post-place*) to make a bipartite graph (Petri, 1966). Edges are directed and have weights associated with them. In biological systems, such as BRNs, places (○) represent biological entities e.g., protein or their complexes, gene, mRNA, ions, metabolites and cell or cellular components while transitions (□) represent biochemical reactions e.g., association, activation, decomposition, inhibition, phosphorylation, dephosphorylation and translocation (Tareen and Ahmad, 2015). The weights of edges represent the stoichiometry of reactions. The weight can be one (1) or greater than one (Blätke et al., 2011; Liu et al., 2016). Following are the different types of edges.

- **Standard edge(→)** is use to represents a simple biochemical reaction such as synthesis, decomposition, replacement and activation reactions. It is enabled when pre-places have adequate tokens. When the transition is fired, tokens from pre-places are removed and then deposited in post-places according to arc weights.
- **Inhibitor edge**(—◦) connects only a pre-place to a transition. If the corresponding pre-place is not adequately marked i.e., it has less tokens than weight of arc, then, the transition is enabled otherwise, it is un-firable. Tokens are not removed from pre-place in the inhibitor edge.
- **Read edge (—•)** connects only a pre-place to a transition. It enables the transition when the corresponding place is adequately marked. The tokens of a place are not altered when the transition is fired.
- **Equal edge (–•−•)** connects pre-place to transition. It may fire a transition when number of tokens in a pre-place is equal to corresponding arc weight. After firing of transition, tokens are not removed from the respective pre-place.

The following definitions are originally given in David and Alla (2010).

**Definition 1** (Unmarked Petri Net). *An unmarked Petri Net (PN) is a five-tuple* ℙ = 〈P,T,E,𝔭𝔯𝔢,𝔭𝔬𝔰𝔱〉 *where:*

- P *is a finite set of places i.e.*, P *= {*$P_{1}$, $P_{2}$, $P_{3}$*…*, $P_{n}$*}*,
- T *is a finite set of transitions i.e.*, T *= {*$T_{1}$, $T_{2}$, $T_{3}$*, …*, $T_{n}$*}*,
- P∩T*=* ∅*, both* P *and* T *are non-empty sets*,
- E⊆P×T∪T×P*, is a set of input and output edges of transitions*.
- 𝔭𝔯𝔢 *:* P×T→ ℕ *, is a weight function that assigns non-negative integers to input edges*.
- 𝔭𝔬𝔰𝔱 *:* T×P→ ℕ *, is a weight function that assigns non-negative integers to output edges*.

**Figure 7:**
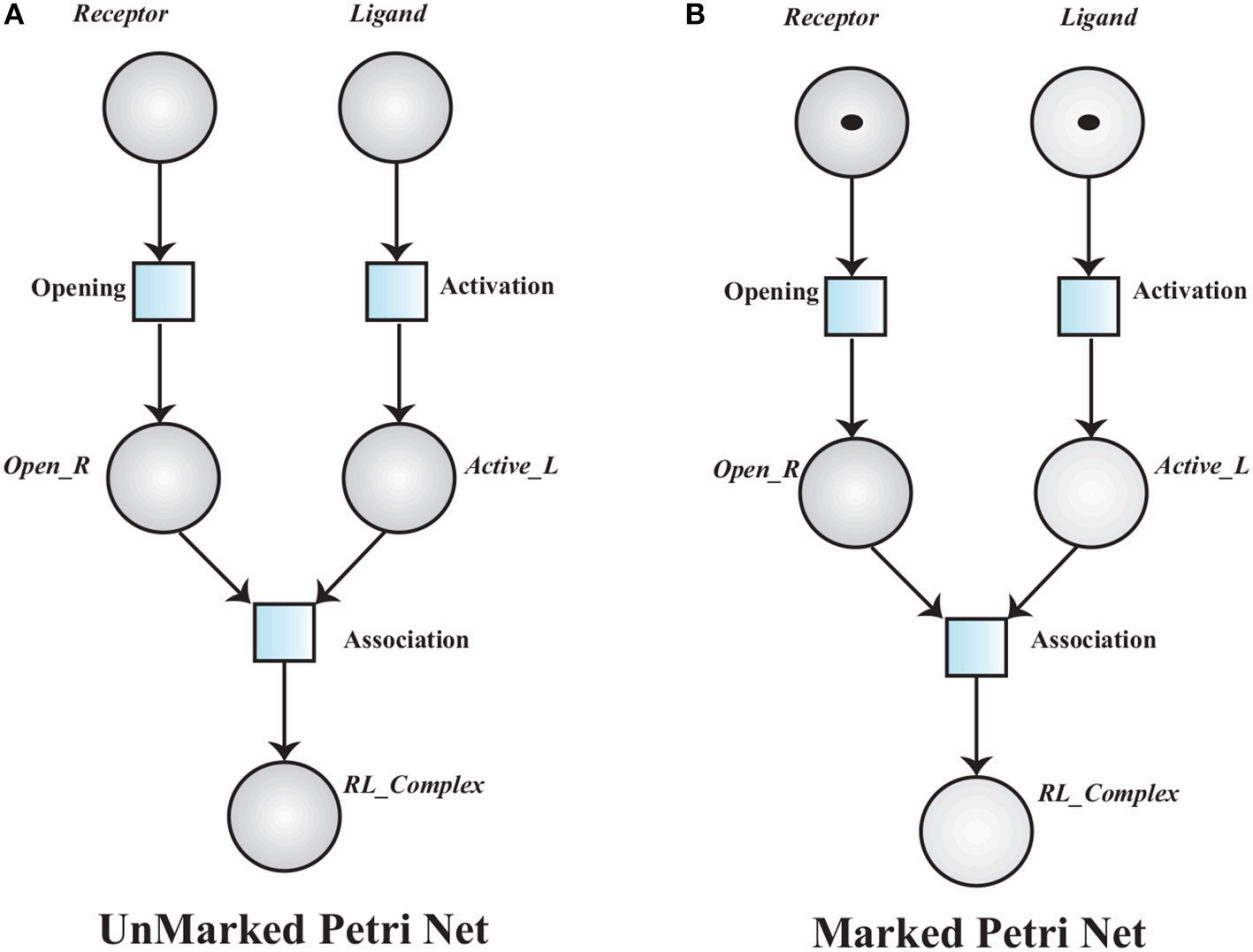
**(A)** An Unmarked Petri net model of Receptor Ligand binding. *RL*_*Complex* is formed when *Receptor* is in open conformation (*Open*_*R*) and *Ligand* is activated into *Active*_*L*. **(B)**, A marked PN model has tokens which represent initial marking of the Receptor Ligand binding model.

A simple unmarked PN is given in Figure 7A, to model a receptor-ligand association. A receptor is in closed conformation and ligand is in in-active form. The receptor must undergoes open conformation and then the ligand activates to form an association (*RL*_*Complex*) with receptor. The places in a PN may have tokens (black dots or positive real numbers) which represent marking of the places. In a marked PN, places are initially assigned tokens.

**Definition 2** (Marked Petri Net). *A Marked Petri Net (MPN) is a tuple* 𝕄ℙ = $\langle \mathbb{P},\overset{ˇ}{{𝔪}_{0}}\rangle$ *where:*

- ℙ = 〈P,T,E,𝔭𝔯𝔢,𝔭𝔬𝔰𝔱〉 *is an unmarked PN and*
- $\overset{ˇ}{{𝔪}_{0}}$ *:* P→ ℤ_≥0_*, is an initial marking of the PN*.

The transition associated to a marked place is said to be enabled and it will be fired when the corresponding pre-places have tokens equal or greater than the weight of the associated arc. Receptor opening requires a *Receptor* and ligand activation depends on the presence of an inactive *Ligand*. An active ligand and an opened receptor form a Receptor-Ligand Complex (Figure 7B).

**Definition 3** (Timed Petri Net). *A Timed Petri Net (TPN) is a tuple* 𝕋ℙ = $\langle 𝕄\mathbb{P},\overset{ˇ}{𝔣𝔫}\rangle$ *where:*

- 𝕄ℙ = $\langle \mathbb{P},\overset{ˇ}{𝔪}\rangle$ *is a MPN*.
- $\overset{ˇ}{𝔣𝔫}$ *:* T→ ℝ^+^ *is a function that associates a positive real value i.e., time delay to each transition*.

**Figure 8:**
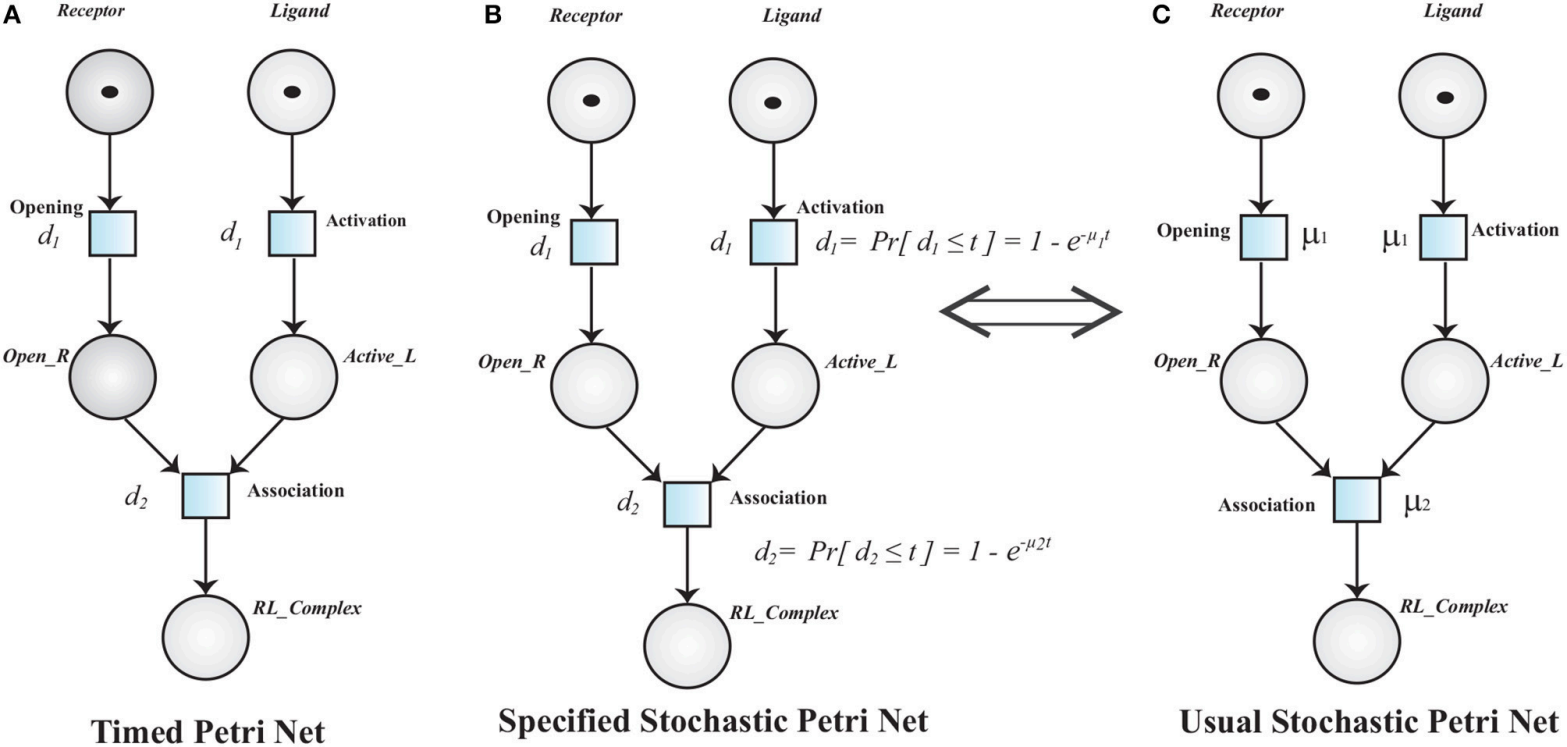
**(A)** A Timed Petri net model of Receptor Ligand Binding. Initially, the *Receptor* is in close conformation and the *Ligand* is inactive. After enabling of respective transition (*Opening* and *Activation*), *Receptor* adopts open conformation (*Open*_*R*) and *Ligand* is activated into *Active*_*L* respectively. The transition *Opening* and *Activation* have an associated time delay *d*_1_. The transitions are fired when the associated time delay is elapsed. *RL*_*Complex* is formed when *Open*_*R, Active*_*L* are present and time *d*_2_ is elapsed. **(B,C)** are Specified and Usual Stochastic Petri Nets respectively. The TPN can be converted into SPN when the deterministic firing function *d* changes into a random variable.

A TPN associates a time delay *d*_*i*_ to a transition $T_{i}$. The transition $T_{i}$ is said to be enabled when it has sufficient tokens in its pre-places and it is fired when its deterministic delay time is elapsed (Figure 8). Stochastic Petri Net (SPN) are used when time delays are random variables. SPN (Figure 8C) (Marsan et al., 1994; Heiner et al., 2008) are explicitly derived from TPN (Figure 8A).

**Definition 4** (Stochastic Petri Net). *A marked stochastic petri net is a pair* 𝕊ℙℕ = 〈𝕄ℙ, ℜ𝔞𝔱𝔢〉 *where:*

- 𝕄ℙ = $\langle \mathbb{P},\overset{ˇ}{{𝔪}_{0}}\rangle$ *is a MPN*.
- ℜ𝔞𝔱𝔢 *:* T→ ℝ^+^*, is a function from the set* T *of transition to the set of finite positive real numbers. ℜ𝔞𝔱𝔢($T_{i}$) = μ_i_, is the firing rate associated with transition $T_{i}$*.
  *The random variables d_i_ have assigned negative exponential probability distribution function (PDF) $\overset{ˇ}{𝔣𝔫}$(t)*,
  $$\begin{array}{l} \overset{ˇ}{fn}(t)=Pr[d_{i}\leq t]=1-e^{-\mu_{i}t}\;where,\\ \;Pr[d_{i}\leq t+dt|d_{i}>t]=\;\mu_{i}\cdot dt \end{array}$$

The marking of $\overset{ˇ}{{𝔪}_{t}}$ of SPN is a homogenous Markovanian Chain (HMC) process, which is a class of stochastic processes simply built from the reachability graph of a qualitative PN by assigning transition rates to edges between all the states (Heiner et al., 2008). Thus an HMC can be associated with every SPN. Gillespie was the first one who designed special case of Petri nets for reaction networks and called them SPNs (Gillespie, 1976). Snoopy (Marwan et al., 2012) tool is used to design, animate and simulate SPNs of neurodegeneration related BRNs. This tool has been extensively used to model a variety of systems such as software systems, biological systems and production systems. The supplementary file S1 contains a general Enzyme-Substrate BRN that is modeled and simulated through the Petri net modeling tool Snoopy. This file helps to explain the working of the tool (Snoopy).

## 3. Results

In this study, stochastic modeling and analysis of the neuronal pathways involved in neuro-degradation in AD was carried out. The SPN models of the three main neuronal physiological pathways: Calcium Influx Efflux channels (Figure 5), Calpain-CAST regulatory system (Figure 4) and APP digesting pathways (Figure 2). The SPN model of the crosstalk of these three pathways is also presented.

### 3.1. SPN of calcium influx efflux channels

**Figure 9:**
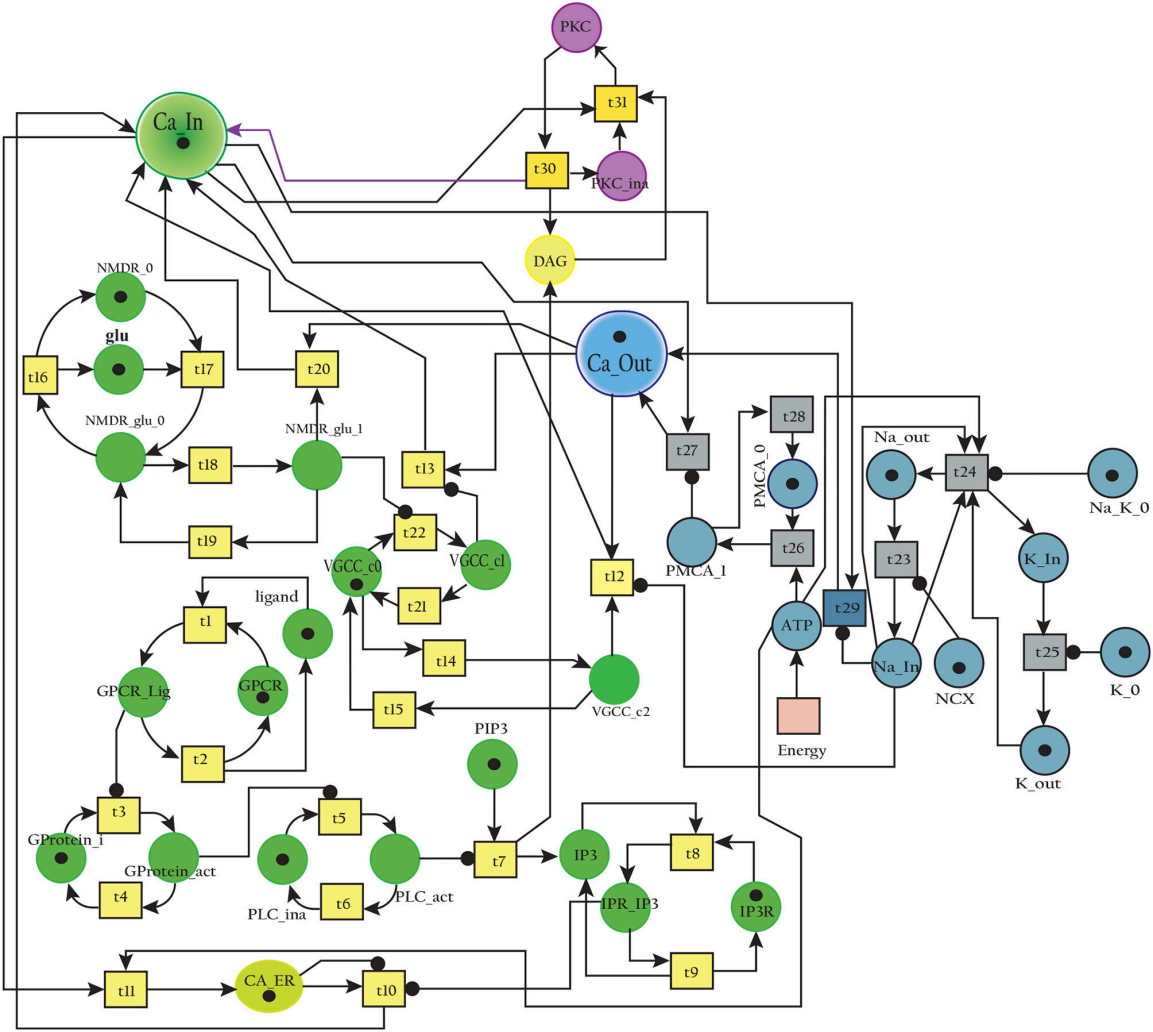
SPN model of the Calcium Channel.

**Figure 10:**
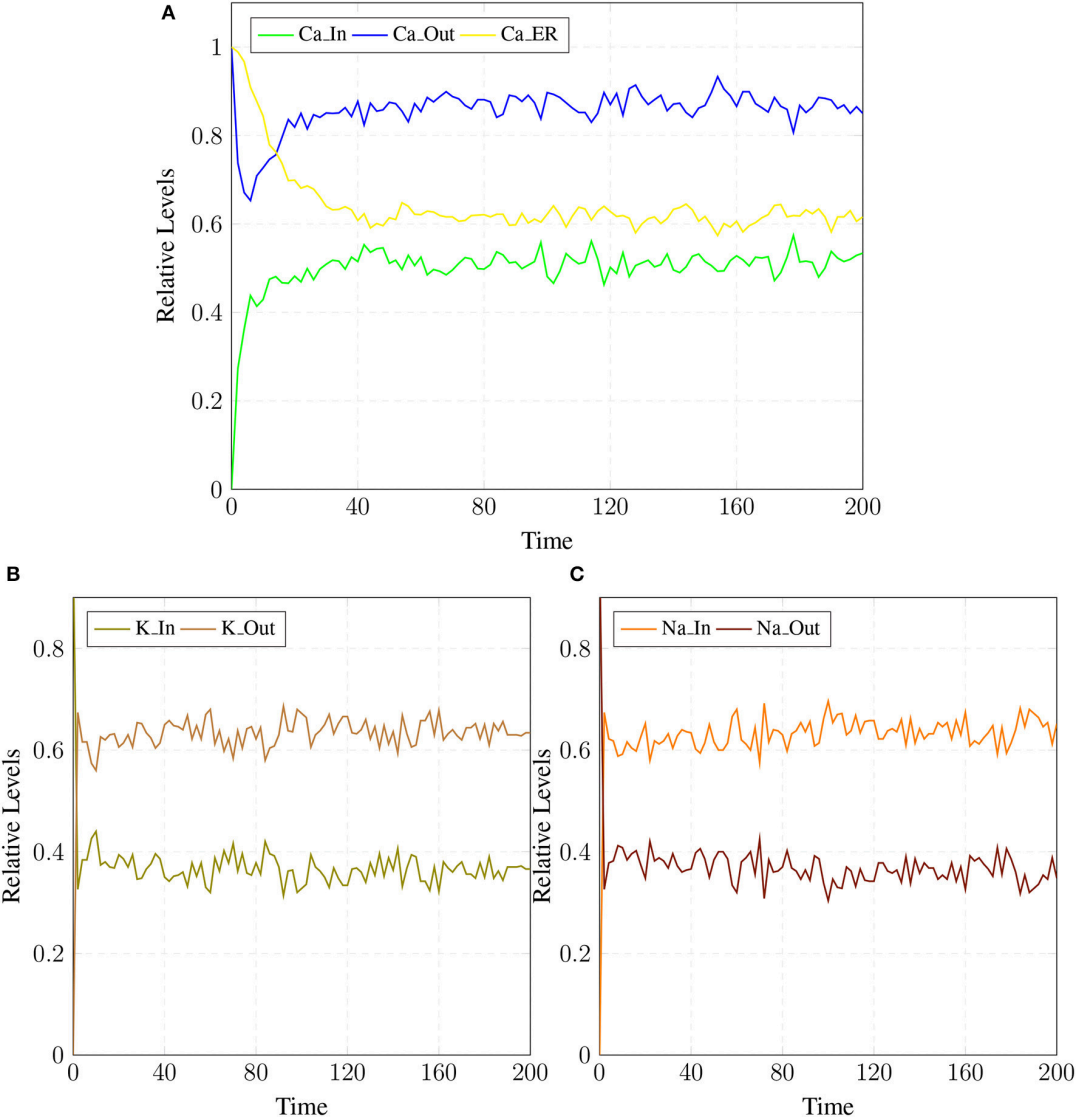
**(A)** shows Calcium homeostasis which is maintained by influx (***Ca***_***In***), efflux (***Ca***_***Out***) and intracellular storage (***Ca***_***ER***). Calcium efflux is further maintained by coordinated and controlled *in and out* flow of Potassium (***K***_***In***, ***K***_***Out***) in **(B)** as well as of the Sodium (***Na***_***In***, ***Na***_***out***) ions channels **(C)**.

The BRN of Calcium influx efflux channels in Figure 5 is translated into a SPN model as shown in Figure 9. The influx channels comprise of receptor based and voltage gated channels (NMDR, VGCC1 / VGCC2). In addition, the GPCR channel regulates intracellular trafficking of Ca^2+^ ions and stimulation of ***PKC*** signaling pathway. The energy dependent efflux channels (e.g., PMCA and Na-K ATPase) work efficiently in a sync with influx channels to maintain equilibrium between in-out flow of Ca^2+^ ions in neurons. Ca^2+^ ions move from ***Ca***_***In*** place (green) to ***Ca***_***Out*** place (blue) and vice versa. The places in green colors and transitions (yellow) constitute influx channels. The places in light blue colors and transitions in grey colors make efflux channels. Initially, ***NMDR***_***0*** place represents that receptor is in close conformation that binds with ***glu*** to form the complex represented by ***NMDR***_***glu***_***0*** place. This complex adapts open conformation represented by the place ***NMDR***_***glu***_***1*** and then further triggers the opening of VGCC channel represented by the place ***VGCC***_***c1*** to facilitate Ca^2+^ ions influx. Excess Ca^2+^ ions are deposited into the place ***Ca***_***ER*** through store-operated channel. The place ***GProtein***_***i*** represents inactive GPCR, that change into place ***GProtein***_***act*** by a complex of GPCR and ligand represented by places ***GPCR***_***Lig*** (complex), ***GPCR*** (receptor) and ***ligand*** respectively. ***GProtein***_***act*** activates PLC represented by place ***PLC***_***act***, which mediate cleavage of ***PIP2*** place into ***DAG*** and ***IP3***. The place ***DAG*** activates ***PKC*** by adding Ca^2+^ ion in it. At the surface of ER, IP3 binds with its receptor IP3R, represented by places ***IP3*** and ***IP3R***, which induces Ca^2+^ ions flow into the place ***Ca***_***ER***. At the same time, Ca outflow is maintained by PMCA, NCX and Na-K ATPase. PMCA channel represented by place ***PMCA***_***1*** requires ATP (place ***ATP***) to extrude one Ca^2+^ ion into extracellular space. The place ***NCX*** representing NCX pump ensures extrusion of one Ca^2+^ ion by adding three Na ions into the cytoplasm represented by place ***Na***_***In***. The place ***Na***_***K***_***1*** represents active (i.e., depends on ATP) Na-K ATPase pumping that pumps Na out of the neuron (place ***Na***_***out***) and K into the neuron (place ***K***_***In***). The place ***VGCC***_***c2*** representing Ca inflow is also dependent on place ***NCX***, which depolarizes the membrane. The SPN model is simulated after applying and adjusting the rates of transitions in order to reproduce physiological working of the pathway. The transitions are labeled by *t*, transition from ***t1***–***t22*** are associated with influx channels. ***t1***–***t10*** are linked to GPCR channel which help in storage of excess Ca^2+^ ions into place ***Ca***_***ER***. ***t11*** takes Ca^2+^ ions from ***Ca***_***ER*** into cytoplasm (place ***Ca***_***In***). Remaining ***t12***–***t22*** make NMDR and VGCC channels functional. The efflux channels, consisting of NCX, NKA channels and PMCA are connected with transitions ***t23***–***t29***. The transitions ***t30*** and ***t31*** are involved in activation of PKC which is dependent on calcium. The rates of transitions are shown in parameter in Table 1. The simulation results have shown that there is an equilibrium behavior in all channels, which is required to maintain homeostasis in calcium flow. In Figure 10A, a stable oscillating behavior can be observed among the places ***Ca***_***out***, ***Ca***_***ER*** and ***Ca***_***In***. Concentration of Ca^2+^ ions in ***Ca***_***out*** place is higher while it is lower in place ***Ca***_***In***. Na and K ions, (Figures 10B,C) are also oscillating properly, contributing to Ca^2+^ homeostasis and generating nerve impulses through polarization and depolarization. Ca^2+^ homeostasis has pivotal role in cell physiological working. Dysregulation of Ca^2+^ homeostasis will affect the regulation of most of the proteins, enzymes and genes which will have deleterious effects on the physiological processes.

**Table 1:** Transition rates of the SPN model of Calcium Channel.

**Tansitions**	**Rate (μ)**	**Transitions**	**Rate (μ)**
*t*1	1	*t*17	1
*t*2	1	*t*18	1
*t*3	1	*t*19	1
*t*4	1	*t*20	1
*t*5	1	*t*21	1
*t*6	1	*t*22	1
*t*7	1	*t*23	1
*t*8	1	*t*24	1
*t*9	1	*t*25	1
*t*10	0.65	*t*26	1
*t*11	1	*t*27	1
*t*12	1	*t*28	1
*t*13	1	*t*29	1
*t*14	1	*t*30	1
*t*15	1	*t*31	1
*t*16	1	*Energy*	1

*The transitions (t) from (Figure 9) are listed with their rates. The transitions are adjusted to rates which can reproduce the physiological working of calcium channels*.

### 3.2. SPN of calpain-CAST regulatory system

**Figure 11:**
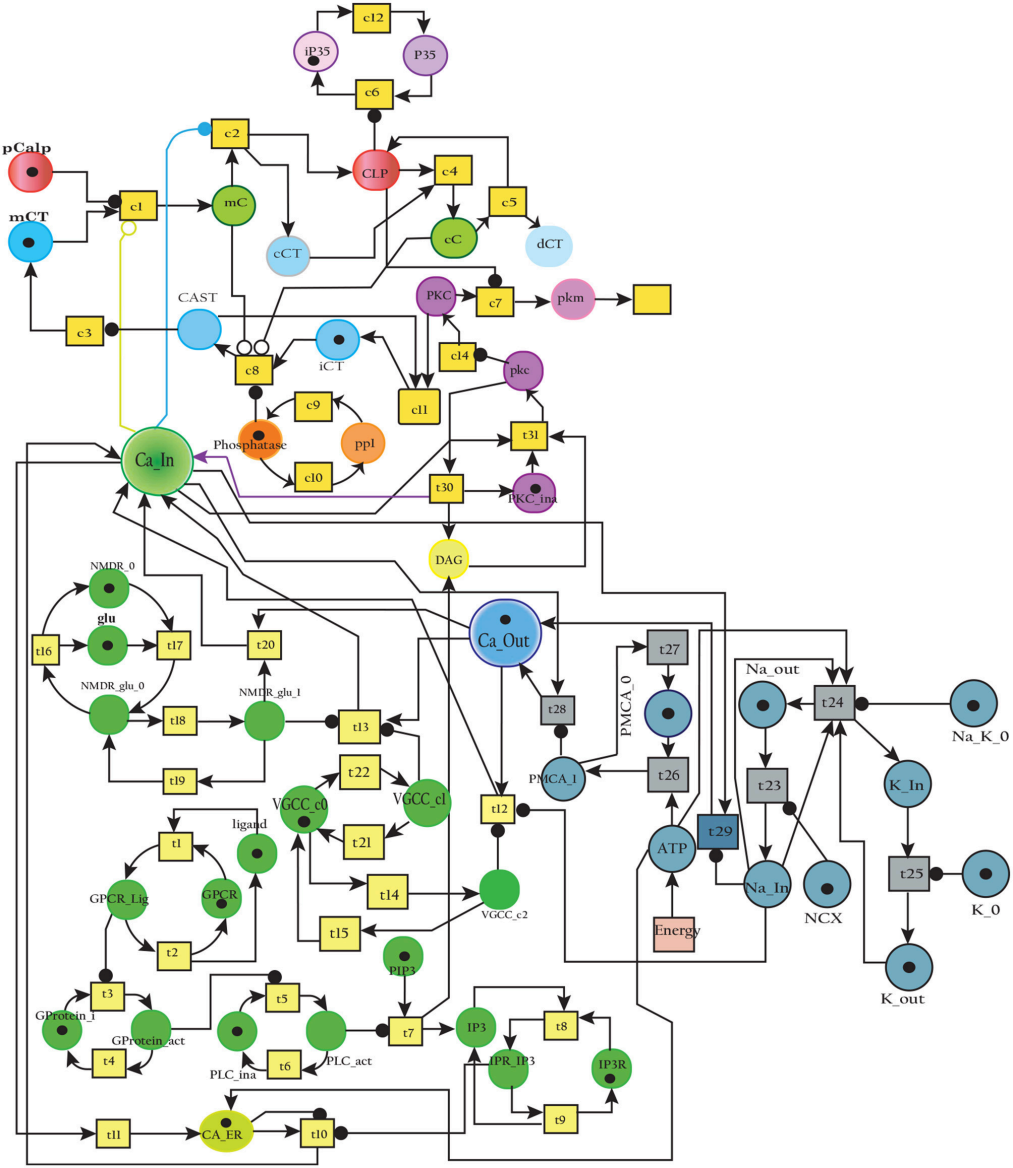
The SPN model of Calpain-CAST system.

**Figure 12:**
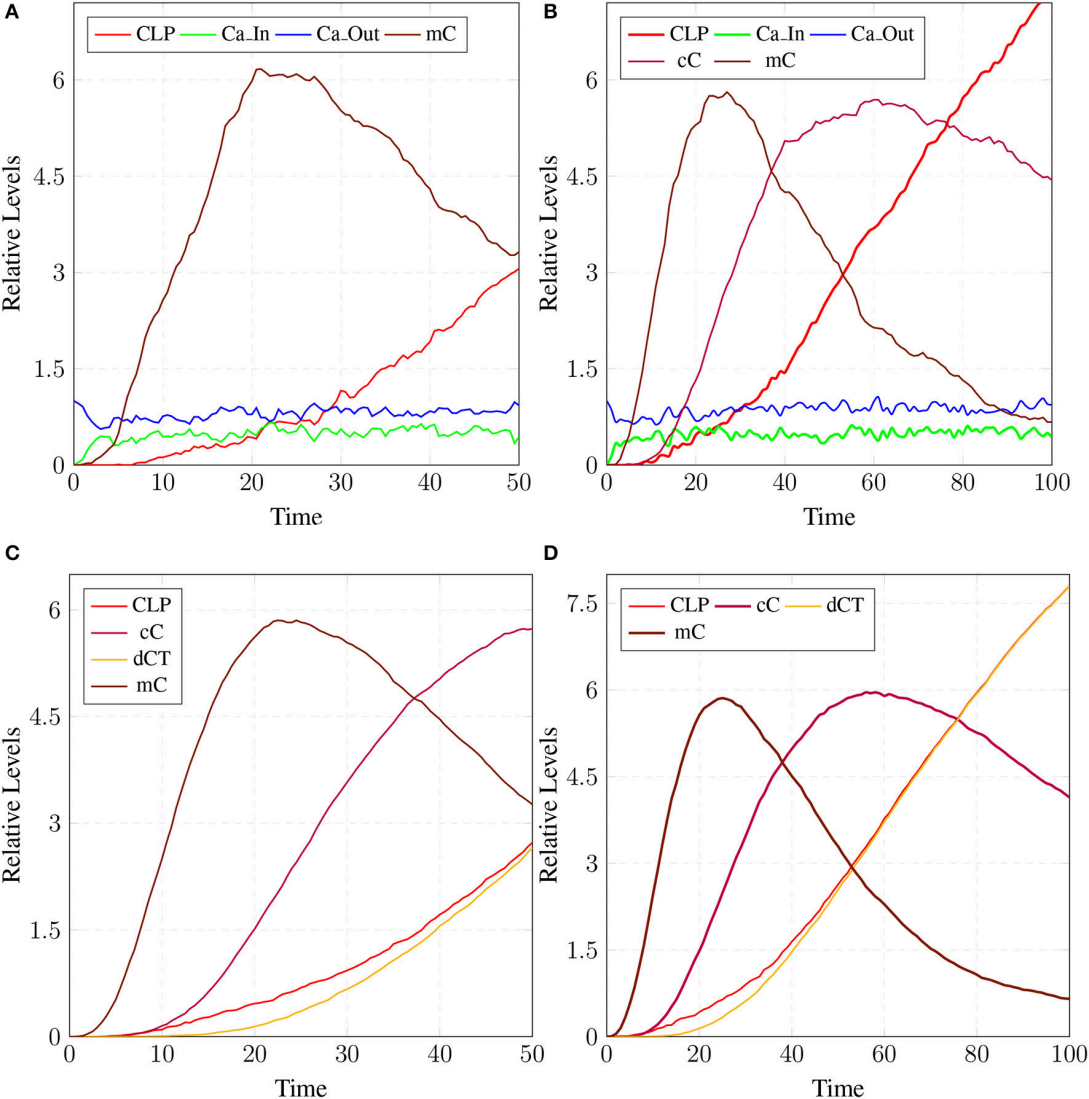
Calpain activation and regulation: In **(A)**, ***CLP*** is bound to CAST in the form of ***mC*** at membrane. As the ***Ca***_***In*** increases and ***Ca***_***Out*** decreases the complex ***mC*** breaks down. In an elaborate view **(B)**, ***CLP*** is produced by the break down of the ***mC*** which is again controlled by the formation of ***cC***. In **(C)**, ***mC*** is formed, maintained and eventually degraded into CAST and Calpain. Both Calpain and CAST form complex ***cC*** which decreases the availability of free ***CLP***. *CLP* binding to ***cC*** slowly degrades the CAST which is represented as ***dCT***. **(D)** Both complexes ***mC*** and ***cC*** are formed while ***cC*** is more stable than ***mC***, as rate of degradation of ***mC*** is higher than **(C)**.

**Figure 13:**
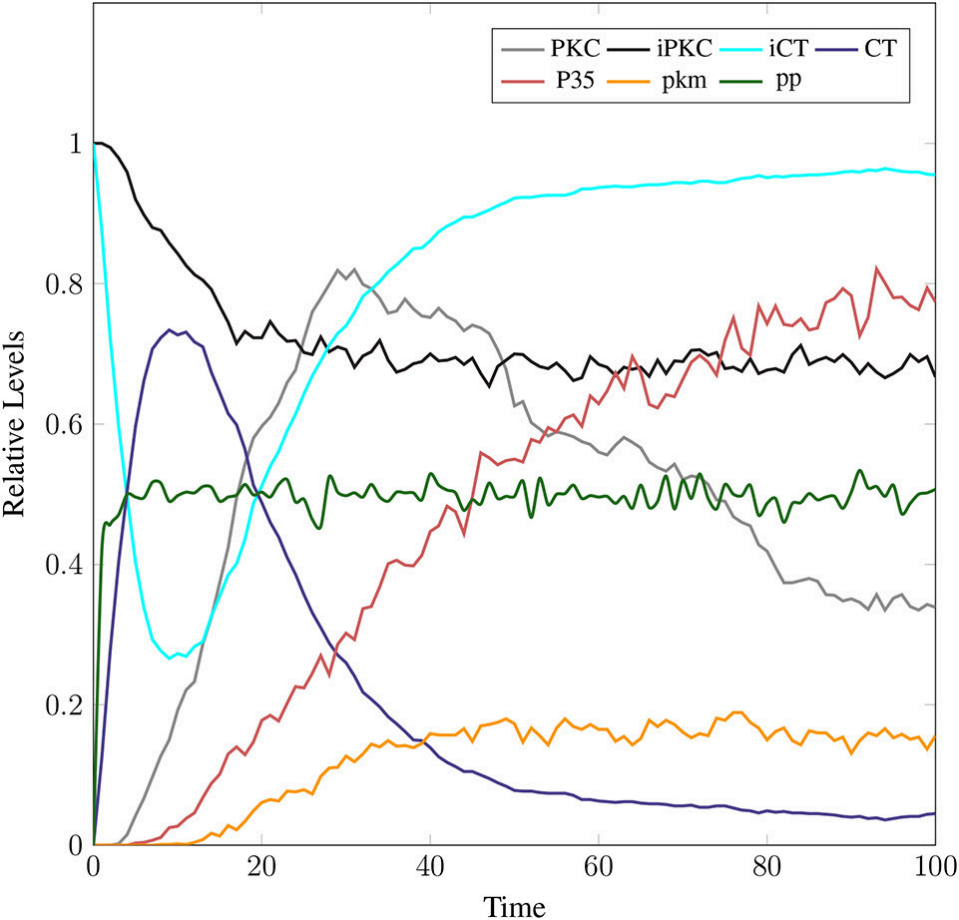
In physiological condition, the helping enzymes (***PKC*** and ***phosphotases***) are periodically activating and deactivating. ***iPKC*** is the dormant form of the cytosol; it activates into ***PKC*** to convert ***mCT*** and ***cCT*** into ***iCT***. Phophotases (***pp***) convert the ***iCT*** into ***CT***. ***PKC*** in the presence of *CLP* is degraded into ***pkm***.

The model in Figure 11 represents Calpain-CAST regulatory system as given in Figure 4. In SPN model, due to lower Ca^2+^ level in cytosol, Calpain is in dormant form which is represented by place ***pCalp***. It then attaches to CAST located on membrane (place ***mCT***) to form a reversible complex of CAST and Calpain (place ***mC***). Calpain is activated when cytosolic Ca^2+^ rises to specific concentration (Pal et al., 2001; Ryu and Nakazawa, 2014). Lower level of cytosolic Ca^2+^ represented by place ***Ca***_***In*** facilitates (place ***mC***), membrane bound inactive reversible Calpain-CAST complex. After the concentration of ***Ca***_***In*** rises or crosses its threshold, then the short-lived ***mC*** is disintegrated into places ***CLP*** and ***cCT***. The place ***CLP*** shows that Calpain moves to transmembrane and then converts into active form in the presence of Ca^2+^ ions and its autolysis. Now place ***CLP*** shows that Calpain is activated that is free to translocate to cytosol. The place ***cCT*** represents that CAST can again hinder over-activation of ***CLP*** by forming a reversible complex, represented by place ***cC***. The place ***cC***, splits into places ***CLP*** and ***dCT***. In nature, active Calpain breaks down the bounded CAST into small subunits (Rao et al., 2008). The place ***CLP*** shows that it is active and which can be involved in various cellular processes. The place ***CLP*** can cleave place ***P35*** into *p25* which is involved in hyper-activation of cyclin dependent kinase 5 (*cdk5*) (Kusakawa et al., 2000; Lee et al., 2000). The transitions of this system are labeled with *c*. The transition ***c1*** forms complex, place ***mC*** from ***pCalp*** and ***mCT*** at low Ca^2+^ concentration. The ***c2*** breaks complex and activates ***CLP*** when level of Ca^2+^ rises into cytoplasm. The transition ***c4*** forms complex place ***cC*** by binding ***CT*** and ***CLP*** in cytoplasm and ***c5***, at a very low rate (Table 2) slowly degrades the CAST into ***dCT*** and makes the ***CLP*** free. Transitions ***c6***, ***c12*** and ***c13*** are linked to ***CLP*** mediated ***P35*** pathway. ***CLP*** also degrades ***PKC*** into ***pkm*** through ***c7***. Remaining transitions ***c3***, ***c8***, ***c9***, ***c10***, ***c11*** and ***c14*** are associated to CAST regulation through ***PKC*** and phosphotases ***pp***. The Table 2 lists all reaction rates of associated transitions which are obtained after fine tuning. In Figure 12A, the simulation shows that when Ca influx, ***Ca***_***In***, is lower than its threshold then ***pCalp*** is bounded into complex of Calpain-CAST (***mC***) at membrane. When ***Ca***_***In*** crosses its threshold, the peak of ***mC*** runs down and ***CLP*** rises representing release and activation of Calpain, (Figure 12B). These results are according to experimental findings (De Tullio et al., 1999; Todd et al., 2003) (Figures 12A,B). To regulate ***CLP*** activation, ***cC*** appears but gradually the complex peak falls down showing that the ***cC*** is broken down into active ***CLP*** and ***dCT***. The results in Figures 12C,D show that as ***CLP*** is rising, both the complexes (***mC*** and ***cC***) are gradually degrading causing depletion of CAST from the cytosol (Averna et al., 2001b; Tompa et al., 2002). In Figure 13, regulation of ***PKC***, CAST and phophatases (represented as ***pp***) can be observed. ***iPKC*** converted into ***PKC*** in the presence of Ca^2+^, which further converts into ***pkm*** by the action of ***CLP***. As shown in the graph of Figure 13, ***CT*** is regulating through means of reversible phosphorylation and dephosphorylation expedited by ***PKC*** and ***pp***, respectively.

**Table 2:** The transition rates of the SPN model of Calpain-CAST regulatory system.

**Tansitions**	**Rate (μ)**	**Transitions**	**Rate (μ)**	**Transitions**	**Rate (μ)**
*t*1	1	*t*17	1	*c*1	0.5
*t*2	1	*t*18	1	*c*2	0.1
*t*3	1	*t*19	1	*c*3	1
*t*4	1	*t*20	1	*c*4	0.02
*t*5	1	*t*21	1	*c*5	0.5
*t*6	1	*t*22	1	*c*6	0.5
*t*7	1	*t*23	1	*c*7	0.3
*t*8	1	*t*24	1	*c*8	0.5
*t*9	1	*t*25	1	*c*9	1
*t*10	0.65	*t*26	1	*c*10	1
*t*11	1	*t*27	1	*c*11	1
*t*12	1	*t*28	1	*c*12	1
*t*13	1	*t*29	1	*c*13	1
*t*14	1	*t*30	1	*c*14	0.5
*t*15	1	*t*31	1	
*t*16	1	*Energy*	1	

*The transitions (t and c) from (Figure 11) are listed with their rates. The transitions are adjusted to rates which can reproduce the physiological working of Calpain-CAST regulatory system*.

### 3.3 APP processing pathways

**Figure 14:**
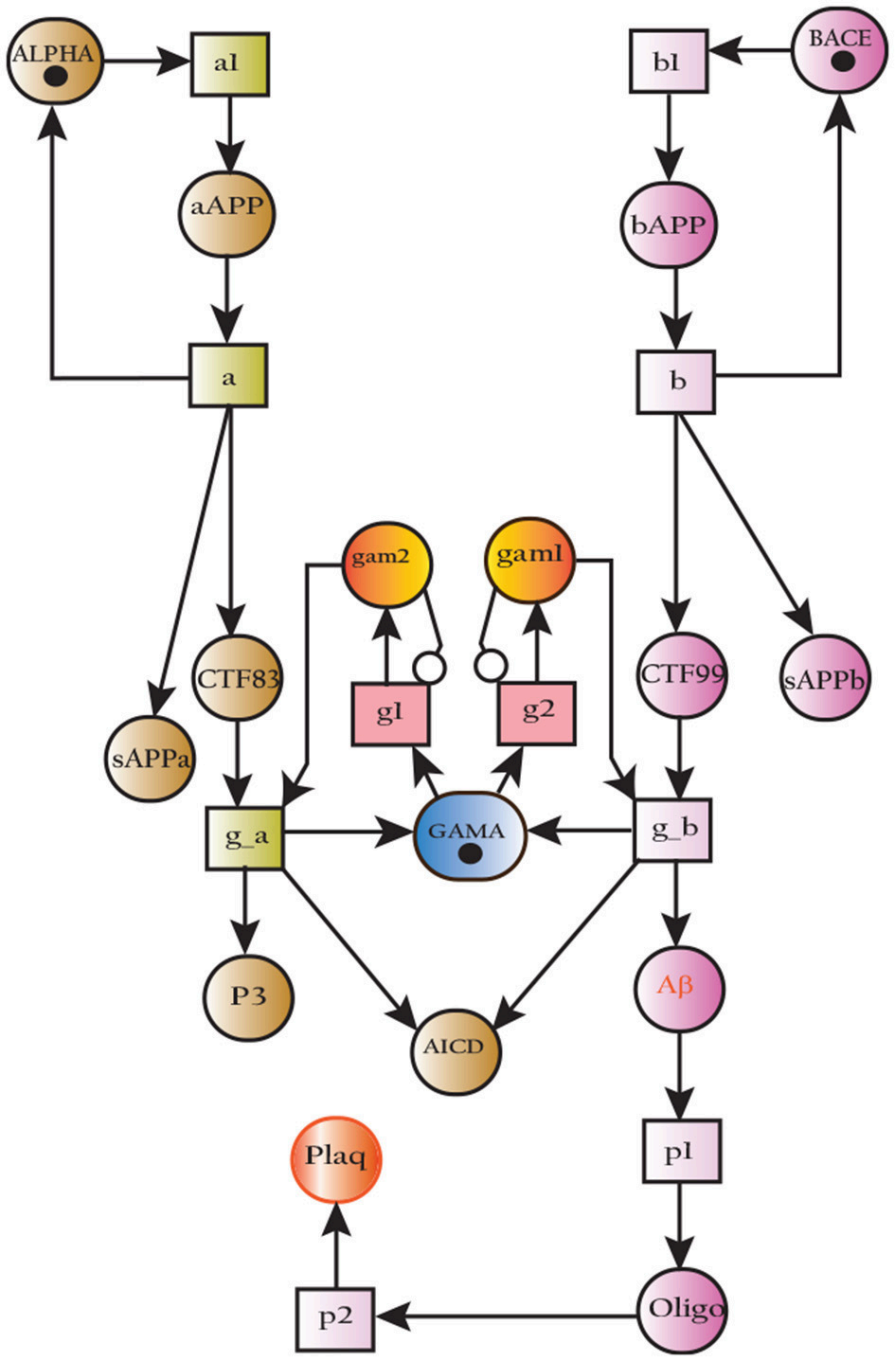
SPN model of non-amyliodogenic (**left**) and amyliodogenic (**right**) of APP processing pathways.

The SPN model of APP processing as shown in Figure 14 is built by combining both amyliodogenic and non-amyliodogenic pathways (Figure 2). APP (place ***aAPP***) is catalyzed by α-secretase (place ***ALPHA***) which produces CTF83 and sAPPα (places ***CTF83*** and ***sAPPa***), whereas digestion of APP (***bAPP***) by β-secretase (place ***BACE***) yields CTF99 and sAPPβ, (represented by places ***CTF99*** and ***sAPPb*** respectively). Further processing is carried out by enzyme γ-secretase (place ***GAMA***) which converts CTF83 into p3 and AICD, while it converts CTF99 into ***A***β and ***AICD***. Aβ accumulate into ***Oligomer*** and finally into ***Plaq***. The transitions ***a***, ***a1*** and ***g***_***a*** constitute non-amyliodogenic pathway, while transitions ***b***, ***b1*** and ***g***_***b*** are linked to amyloidogenic pathway. The parameters are set according to Table 3, in which the rate of transition of α-secretase (place **ALPHA**) mediated APP processing (***aAPP***) is higher than BACE (***BACE***) driven APP digestion (***bAPP***) ***(***μ***= 0.5, 0.3 respectively)***. The rates of plaques formation and accumulation (transitions: ***p1*** and ***p2***) are also adjusted according to the observations in literature which predict low *Aβ* burden in normal healthy brain (Mawuenyega et al., 2010; Rodrigue et al., 2012).

**Table 3:** The transition rates of the SPN model of APP processing pathways.

**Tansitions**	**Rate (μ)**
*a*	0.5
*a*1	0.5
*b*	0.3
*b*1	0.3
*g*1	0.5
*g*2	0.5
*g*_*a*	0.5
*g*_*b*	0.3
*p*1	0.005
*p*2	0.02

*The transitions from Figure 14 are listed with their rates. The transitions are adjusted to rates which can reproduce the physiological working of amyloidogenic and non-amyloidogenic pathways*.

**Figure 15:**
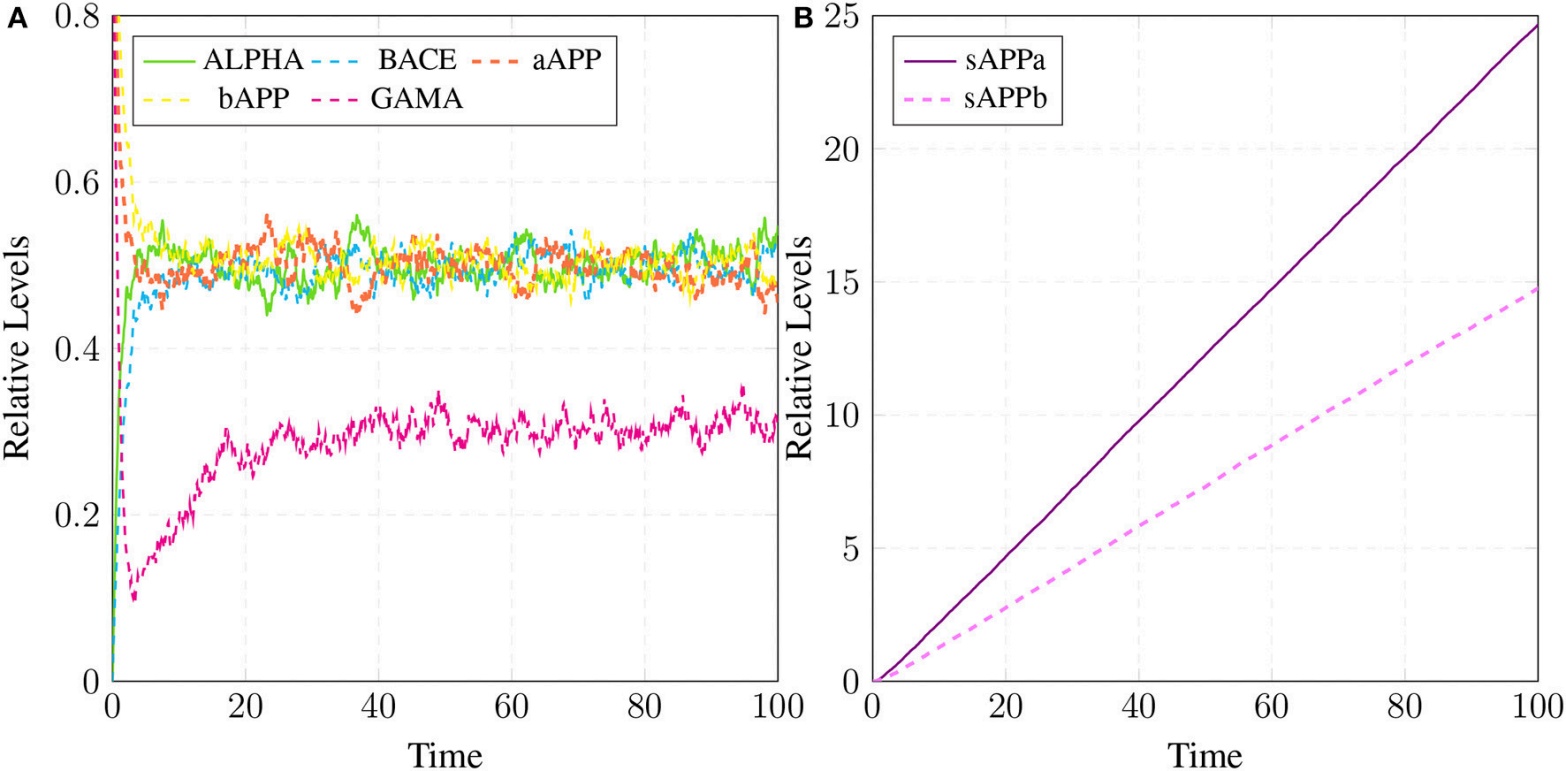
APP processing in physiological conditions: In **(A)**, substrate *aAPP* binds with alpha secretase (***ALPHA***) and ***bAPP*** binds with **BACE**. All the three secretases ***ALPHA,BACE***, and ***GAMA*** are oscillating. In **(B)**, concentration of ***sAPPa*** is higher than the concentration of ***sAPPb*** which depicts healthy cell physiology.

**Figure 16:**
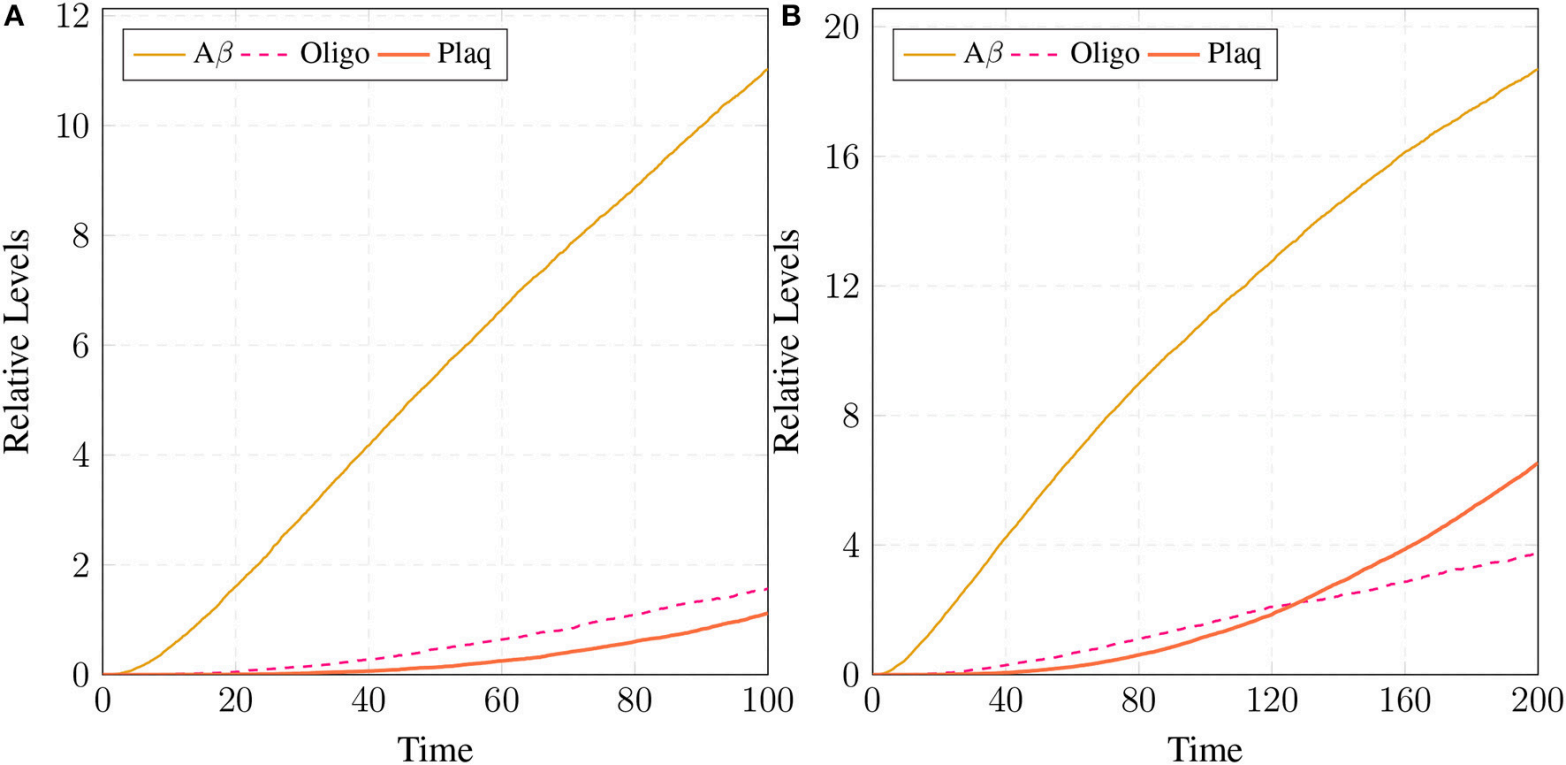
Plaques formation in healthy brain: In **(A,B)** production of plaque is shown over the time period of 100 and 200 unit time, respectively. ***Plaq*** accumulation starts after sixty to eighty unit time. ***A***β and ***Oligo*** show fluctuating behaviors as they start to build-up but degrade with time. First, ***A***β gathers into large quantity to form ***Oligo*** which gradually accumulates into ***Plaq***.

The simulations in Figure 15A shows that all the three enzymes (***ALPHA***, ***BACE***, and ***GAMA***) are available for the proteolysis of APP (in the cell to digest the around-the-clock production of) ***APP***. The Figure 15B shows that the product ***sAPPa*** of non-amyloidogenic processing pathway is in higher concentration than the product ***sAPPb*** of amyloidogenic pathway. Production rate of ***sAPPa*** is higher than ***sAPPb***. The graph in Figure 16A shows that in the healthy brain plaques are produced and accumulated in a linear fashion. *Aβ* burdens in the form of plaques at elder age i.e., approximately after 60 unit to 100 unit time. In Figure 16B, the linear behavior of plaques change to an exponential growth due to the fast accumulation rate with passage of time which depicts the lag phase (no plaques appearance) upto 80 unit time and after it evolves into growth phase (evolution).

### 3.4. Cross talk of calcium channels, APP and calpain-CAST regulatory pathways

**Figure 17:**
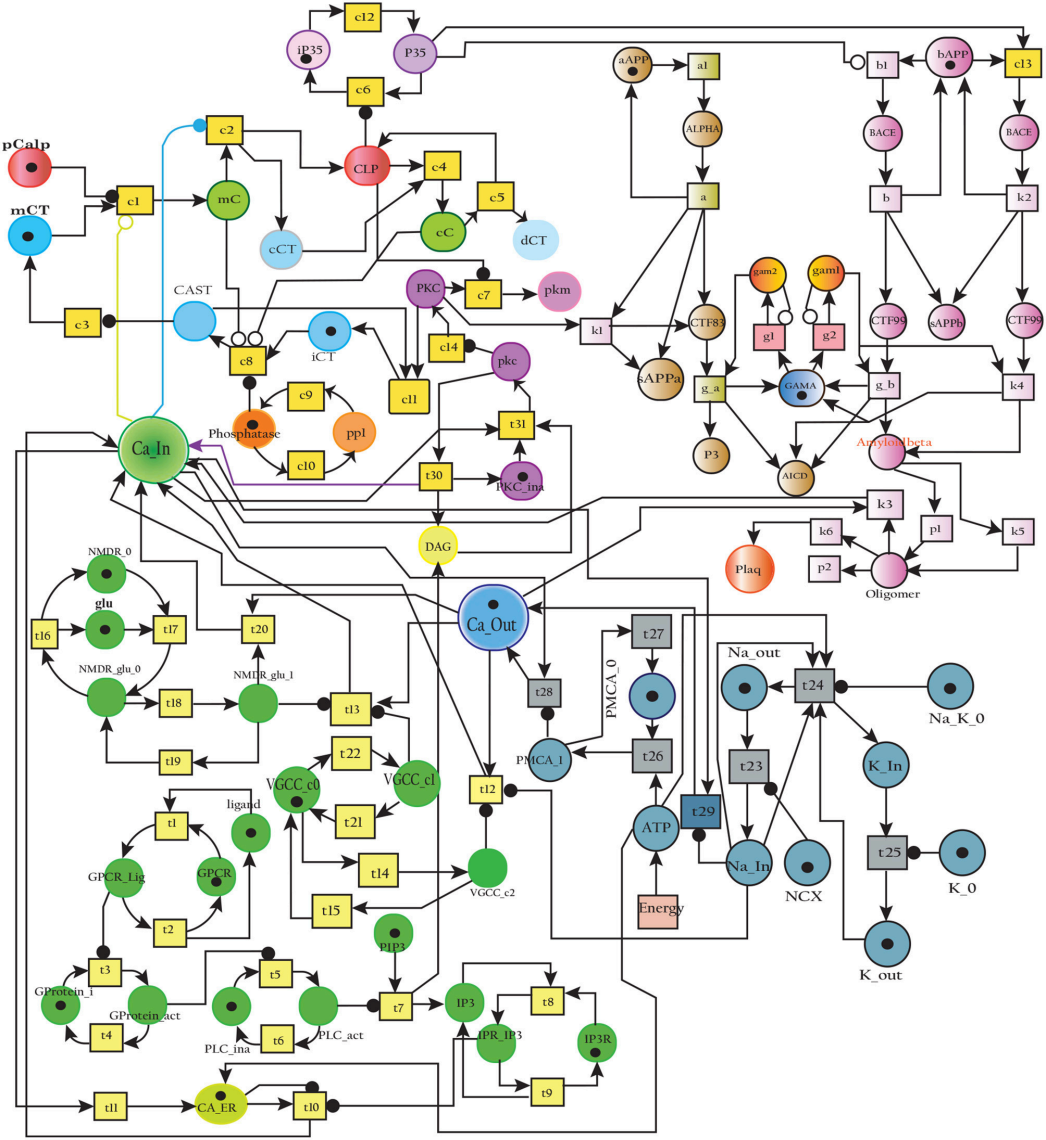
SPN model of the cross talk network of calcium homeostasis, APP processing and Calpain-CAST system Pathways.

The Figure 17 represents the SPN model of the crosstalk network in Figure 6. The connection that joins the pathways is established through places ***CLP***, ***P35*** and ***BACE***. Active Calpain enhances β-secretase mediated APP cleavage twice than normal (Kusakawa et al., 2000; Liang et al., 2010) which initiates early production and accumulation of plaques (Jack et al., 2010; Braak et al., 2011). As a counter action, PKC ***PKC*** plays its role and enhances the activity of enzyme α-secretase (***ALPHA***) (Skovronsky et al., 2000). In the next connection, Calpain (***CLP***) hinders the functioning of ***PKC*** by degrading it into pkm (place ***pkm***). Aβ oligomers represented by place ***Oligomer*** forms pores into the membrane which instantaneously increases influx of Ca^2+^ ions. The place ***Amyloidbeta*** inhibits the transition **Energy** which indirectly hinders Ca^2+^ extrusion into extracellular space. The new transitions in the crosstalk network are labeled with *k*. The transition ***k1*** connects the place ***PKC*** with ***ALPHA*** while ***P35*** is connected to ***Plaq*** through transitions ***c13***, ***k2***, ***k4***, ***k5***, and ***k6***. The transition ***k3*** joins ***Oligomer*** with ***Ca***_***In***. The reaction rates of all the transitions are listed in Table 4.

**Table 4:** The transitions rates of the SPN model of the crosstalk network.

**Tansitions**	**Rate (μ)**	**Transitions**	**Rate (μ)**	**Transitions**	**Rate (μ)**	**Transitions**	**Rate (μ)**
*t*1	1	*t*17	1	*c*1	0.5	*b*	0.3
*t*2	1	*t*18	1	*c*2	0.1	*b*1	0.3
*t*3	1	*t*19	1	*c*3	1	*g*1	0.5
*t*4	1	*t*20	1	*c*4	0.5	*g*2	0.5
*t*5	1	*t*21	1	*c*5	0.02	*g*_*a*	0.5
*t*6	1	*t*22	1	*c*6	1	*g*_*b*	0.3
*t*7	1	*t*23	1	*c*7	0.3	*p*1	0.005
*t*8	1	*t*24	1	*c*8	0.5	*p*2	0.02
*t*9	1	*t*25	1	*c*9	1	*k*1	0.5
*t*10	0.65	*t*26	1	*c*10	1	*k*2	0.6
*t*11	1	*t*27	1	*c*11	1	*k*3	1
*t*12	1	*t*28	1	*c*12	0.5	*k*4	0.6
*t*13	1	*t*29	1	*c*13	1	*k*5	0.01
*t*14	1	*t*30	1	*c*14	0.5	*k*6	0.04
*t*15	1	*t*31	1	*a*	0.5	
*t*16	1	*Energy*	1	*a*1	0.5	

*All the transitions from Figure 17 are listed with their corresponding rate values. As the SPN is a combination of previous three models, it has only three new connections with transition labeled k. All the transitions are adjusted to rates which can reproduce the patho-physiological condition of the brain*.

**Figure 18:**
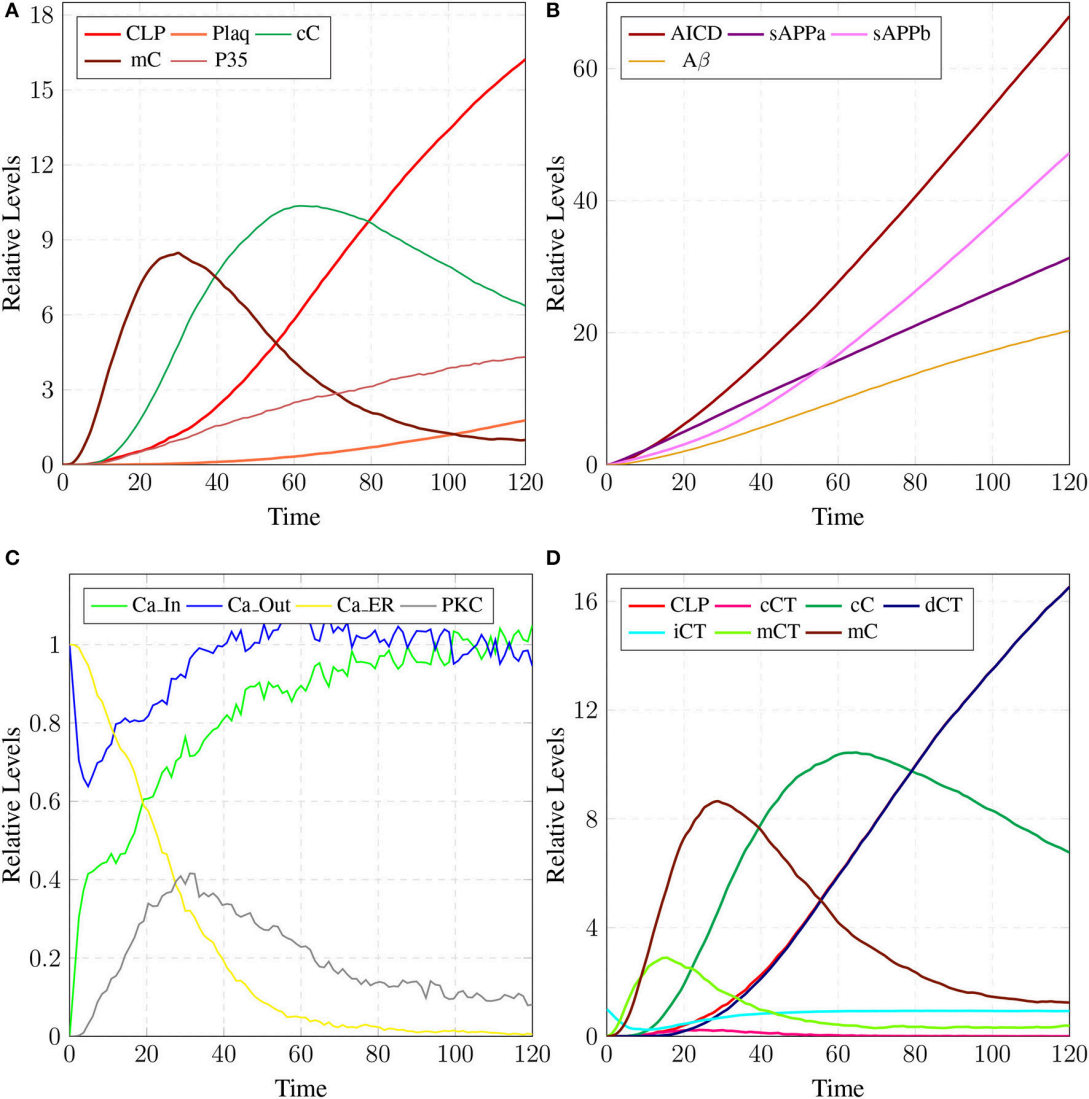
In **(A)**, high concentration of ***CLP*** causes gradual degradation of ***cC*** and further increases the activation of ***P35*** which then activates rapid production of ***Plaq*** leading to the onset of AD. In **(B)**, the production of ***sAPPa*** is lower than the production ***sAPPb*** and ***AICD*** production is higher than both ***sAPPa*** and ***sAPPb***. **(C)** shows slow dysregulation of calcium homeostasis by increase of concentration of ***Ca***_***In*** from ***Ca***_***Out*** and ***Ca***_***ER***. **(D)** shows conversion of ***iCT*** into active form then translocation to membrane ***mCT*** and cytosol ***cCT***. Furthermore, ***cCT*** binds with ***CLP*** to form ***cC***. ***CLP*** gradually degrades CAST into ***dCT***.

The results in Figure 18 show that ***CLP*** and ***P35*** are accumulating which can directly affect the plaques accumulation process by increase in their deposition rate, as compared to results in section 3.3 where accumulation of ***Plaq*** is minimal (Figure 16A). It can also be observed that ***mC*** and ***cC*** start declining at later stages which indicates depletion of CAST (Figure 18A).

In Figure 18B, the concentration of ***sAPPb*** is higher than ***sAPPa*** protein, which is contrary to the Figure 15B. Hyper activity of ***CLP*** influences more than one processes in the pathological network by enhancing ***BACE*** activity which increases the growth of ***sAPPb***. The decrease in ***PKC*** concentration and calcium dysregulation (***Ca***_*In* rises gradually, ***Ca***_*Out* and ***Ca***_*ER* both lower down) are also due to over-activation of ***CLP*** (Figure 18C). Instability in Calcium equilibrium in neurons appear after 60 unit time which also act as a signal indicating the development of AD. Additionally, degradation of ***PKC*** into ***pkm*** by the action of ***CLP*** also have bidirectional effect on the system. First the availability of active ***CT*** in the cell raises and then ***ALPHA*** secretase driven APP processing slows down (Figure 18B). Figure 18D represents the translocation and regulation of CAST (***cC*** and***cC***) in the brain cell.

### 3.5. Therapeutic intervention

**Figure 19:**
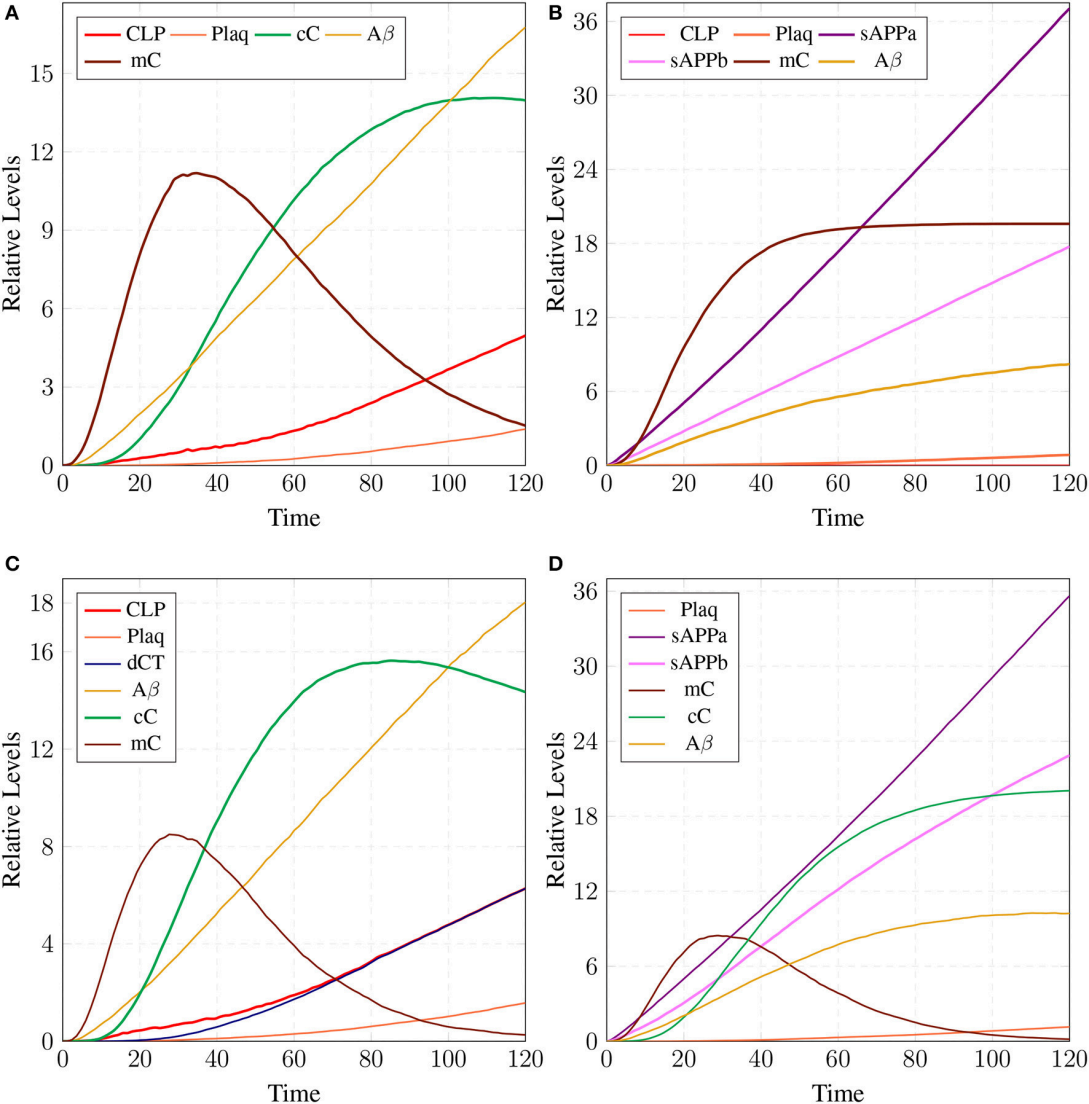
A mild cognitive impairment behavior in **(A,C)**. ***CLP*** is in loosely controlled by ***mC*** and ***cC*** (*c2* = 0.05 and *c5* = 0.005). There is low level of ***Plaq*** and significant level of ***A***β. By changing the rate of *c2* = *0* **(B)** there is no conversion of pro-Calpain into ***CLP*** due to long life of ***mC***. Production of ***sAPPb*** and ***A***β are also decreased sufficiently and it lingers on the process of accumulation of ***Plaq***. In **(D)**, *c5* is set to zero which controls level of ***CLP*** after its activation and saves CAST from depletion into ***dCT***. The production of ***sAPPa*** is higher than production of ***sAPPb*** in **(B,D)**.

The parameter Table 5 shows that by changing the rate of reaction of two crucial transitions i.e., ***c2*** and ***c5*** (representing degradation rate of Calpain-CAST complex at membrane and cytosol, respectively) would provide effective strategy to control or stop the development of AD and other related neurological disorders. After applying *in silico* intervention, it can be observed that as the rate of transition ***c2*** is decreased the neurological network move toward stability due to basal or low activity of ***CLP*** (Figure 19A). Further, the rate of ***c2*** is set to zero which indicates that ***mC*** is made unbreakable or stable which implies that there would be no production of ***CLP*** (Figure 19B). In Figure 19A, both the complexes (***mC*** and ***cC***) are maintained for longer time duration which result in low production of ***CLP***, ***A***β and ***Plaq***. In Figure 19B, there is neither the production of ***CLP*** nor the formation of ***cC*** due to long life of ***mC*** complex occur and there is negligible production of ***A***β. The complex (***mC***) also ensures low level of ***sAPPb*** and high concentration of ***sAPPa*** and as a result there is minor accumulation of ***Plaq*** in the neurons.

**Table 5:** Transition rate table.

**Tansitions**	**Rate (μ)**	**Rate (μ)**
*c*2	0.05	0.0
*c*5	0.005	0.0

*Changing the rate of crucial transitions c2 and c5 to study their effects on crosstalk network model Figure 17*.

**Figure 20:**
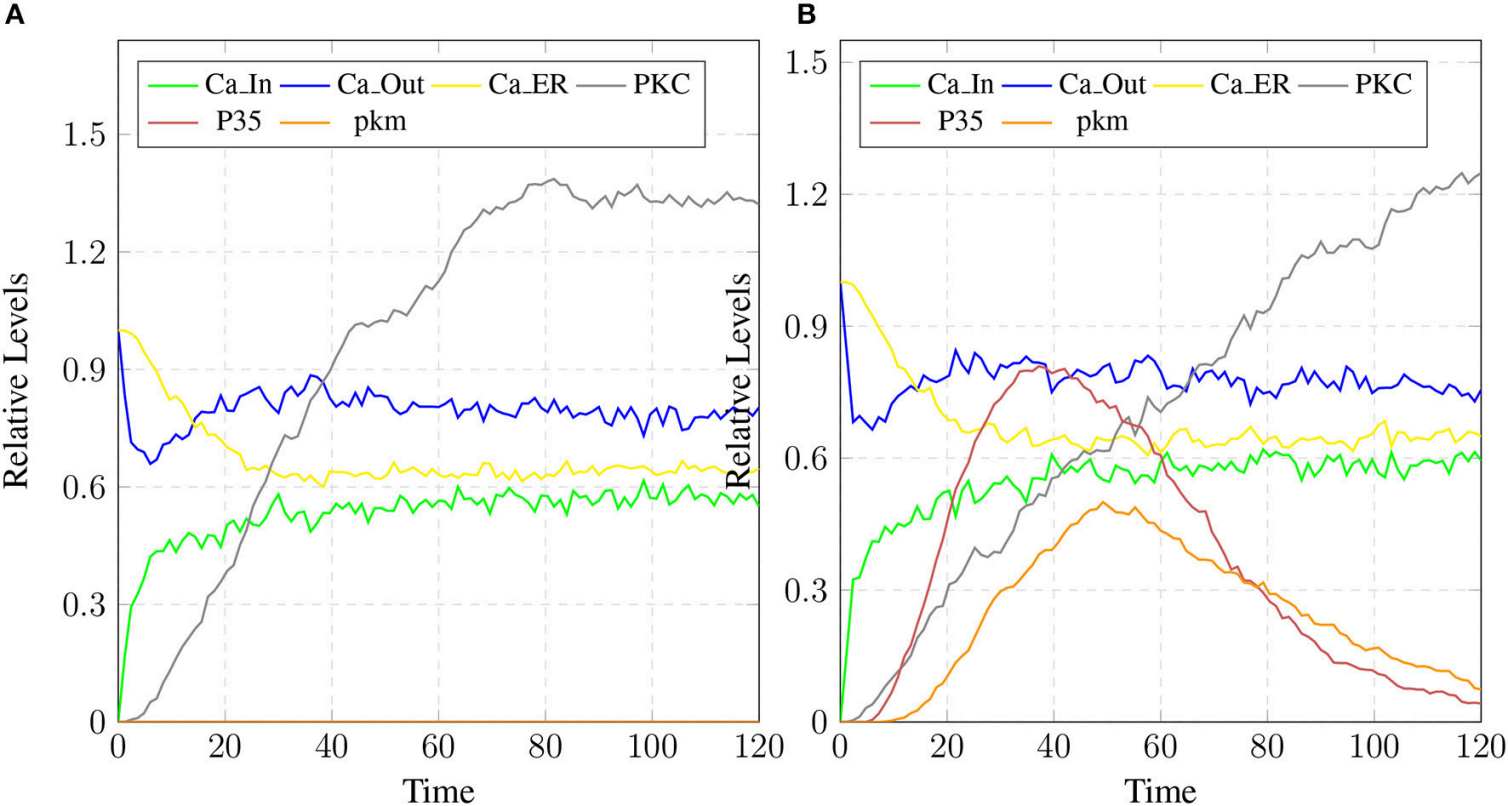
The restoration of Calcium homeostasis. In **(A)**, ***PKC*** is present for longer time and there is no conversion into ***pkm*** also there is no production of ***P35***. In **(B)**, there is less production of ***P35*** and ***pkm***.

The stability of ***mC*** have positive effects in maintaining the calcium homeostasis which regains its equilibrium accompanied by substantial concentration of ***PKC*** and no production of ***P35*** Figure 20A. The smaller rate of ***c5*** also favors stable condition of system and can delay the production of plaques. Figures 19C,D show that as the rate of transition of *c5* decreases the system maintains a stable state due to prolonged life of ***cC*** which causes basal level production of ***CLP*** and eventually guard against rapid ***Plaq*** accumulation in brains. As shown in Figure 19D, ***cC*** helps in maintaining a balanced level of **CAST**, ***sAPPa*** and ***sAPPb*** and it lowers down the concentration and accumulation of ***A***β and ***Plaq***. Also it aids in maintaining homeostasis of other proteins and channels into the cell such as ***PKC***, ***pkm***, ***P35*** and calcium homeostasis (Figure 20B). Therefore, these complexes are very critical targets since they regulate homeostasis of many crucial proteins.

## 4. Discussion

This study contributes in achieving long haul goal of AD research i.e., understanding and manipulating pathological conditions which include BACE and Calpain over-activation, Calcium dysregulation, CAST depletion and abnormal production of Aβ. In this study, neuronal pathways comprising Calpain-CAST system, APP processing pathways and Calcium channels were first modeled individually and then their cross-talk was also modeled. The simulation results of these models predicted a more clearer picture of AD pathogenisis also, on the basis of observations interventions were introduced to linger on the AD pathogenesis.

In a healthy physiological model, rate of Ca^2+^ ions inflow should be lower than rate of Ca^2+^ ions outflow with uniform oscillation within the safest level. Moreover, the concentration of Ca^2+^ ions in intracellular store (***Ca***_***ER***) is greater than (***Ca***_***In***) (Figures 10B,C) (Korol et al., 2008; Gutierrez-Merino et al., 2014). An equilibrated flow is also observed in other important ions channels such as NCX and NKA, which mediate influx and efflux of Na and K ions. Balance in their flow is obligatory for the adequate efflux of Ca^2+^ ions for maintaining homeostasis (Gutierrez-Merino et al., 2014). Another important physiological pathway, Calpain-CAST regulatory system also depends on Ca^2+^. The calcium influx and efflux channels are required for the activation and regulation of Calpain.

The simulation results in section 3.2, show that when concentartion of Ca^2+^ is lower inside the cytosol then Calpain is bounded in the complex of Calpain-CAST at membrane. As concentartion of Ca^2+^ rises, the complex degrades and active Calpain is produced (De Tullio et al., 1999; Todd et al., 2003). To regulate Calpain's activation, CAST appears to form Calpian-CAST complex in cytosol, but gradually its peak falls down which shows release of active Calpain and degradation of CAST. The results in Figures 12C,D show that as ***CLP*** rises, both the complexes (***mC*** and ***cC***) gradually degrade which causes depletion of CAST (Averna et al., 2001b; Tompa et al., 2002). PKC also has important role in building a feedback mechanism for Calpain by phosphorylating CAST into inactive form (Averna et al., 2001a). Calpain is also regulating PKC by cleaving it into pkm (Goll et al., 2003) which is persistently active catalytic fragment and quickly disappears (Yamakawa et al., 2001; Liu et al., 2008). PKC also has important roles in the system, it is favoring α-secretase mediated APP cleavage (Rossner et al., 2001; Racchi et al., 2003) and is also involved in inactivation of CAST through phosphorylation (Olariu et al., 2002). The inactive CAST regains its active conformation in the presence of phosphatases (Averna et al., 2001a).

Simulation results of the model APP processing pathways show that in a healthy brain all three enzymes of this pathway are available in the cell to digest the around-the-clock production of APP with α-secretase having high affinity as compared to BACE (De Strooper and Annaert, 2000). Consequently, production rate of sAPPα is higher than sAPPβ (Figure 15B) which is a depiction of normal physiological behavior (De Strooper and Annaert, 2000; Lichtenthaler, 2011). Studies showed that in healthy brain, plaques produce and accumulate in a linear fashion which is also observed in our studies. Moreover, *Aβ* burdens in the form of plaques at elder age. This linear behavior of plaques accumulation can adopt exponential growth due to the high accumulation rate with passage of time. In our results the formation of plaques first enter the lag phase (no plaques appearance) upto 80 unit time and then evolve into growth phase (evolution), which is also experimentally reported (Friedrich et al., 2010; Jack et al., 2010; Sperling et al., 2011).

In neuro-pathological model, simulation revealed that the intermediate product sAPPβ of amyloidogenic pathway rises in the AD brain. Higher concentration of sAPPβ can lead to early occurrence of AD (at age of 40 and onward) due to rapid deposition of the plaques in brain of AD patients (Freer et al., 2016). Higher level of sAPPβ also indicate the enhanced activity of BACE which is triggered by over-activation of Calpain. Enhanced activation of Calpain disturbs more than one processes in the pathological network i.e., increased BACE activity, decreased PKC functioning and calcium dysregulation. Initially CAST and its complex with pro-Calpain are in high quantity to control Calpain but with the elevation of calcium influx, the complex starts to degrade. The active Calpain accumulates in cytosol which is then controlled by cytosolic CAST by forming complex with it. After sometime Calpain degrades the CAST in the complex to release itself into the cytosol and eventually CAST and complex deplete from the cell (Higuchi et al., 2012). Depletion of CAST destroys the cell normal functioning and then nervous system deteriorates (Rao et al., 2008, 2014; Kurbatskaya et al., 2016).

After analyzing the neuropathological network, the vague picture of AD development becomes more clear. It provide useful observations such as loss of calcium homeostasis occur after 60 unit time due to intra-neuronal ATP depletion caused by elevation of Calpain (Lipton, 1999; Kurbatskaya et al., 2016) and formation of extracellular ion pores formed by Aβ oligomers (Small et al., 2009). Ca^2+^ disruption increases Calpain concentration in neurons which further elevates the BACE activity toward APP (Liang et al., 2010; Chami and Checler, 2012; Song et al., 2015) via cdk5 dependent pathway as a result of Aβ and sAPPβ levels in neurons built up (Sennvik et al., 2004). In normal healthy brain, the mean burden in the form of plaques are low and it increase in linear fashion from 60 to 90 unit time (Rodrigue et al., 2012; Freer et al., 2016; Kurbatskaya et al., 2016). It is noteworthy that both amyloidogenic and non-amyloidogenic pathways are enabled in an healthy individual but plaques in the brain of AD patient grow rapidly due to increased Calpain mediated cleavage of APP through BACE (Mawuenyega et al., 2010). It can be inferred that all the neuro-pathological events are inter-related where Calpain and its complexes are playing crucial role. Calpain can be called as bone of contention and the complexes are the defenders. This neuropathological network provides valuable knowledge about interventions and offers new therapeutic targets. To stop the Calpain destructive effects, it may be effective strategy to slightly modify the natural process. In the work of Emmaneul and coworkers on cardiovascular remodeling, the Calpain-CAST system has emerged as new effective strategy to prevent angiotension (Letavernier et al., 2008). In our study, Calpain-CAST complexes have also proved to be effective therapeutic targets for delaying the process of neuronal degradation which can save the brains from AD.

## 5. Conclusion

In AD, *Aβ* has central role as they accumulate into hallmark lesions i.e., plaques. *Aβ* generates from APP cleavage through amyloidogenic pathway. Progression of the corresponding processing pathway increases due to over-activation of Caplain and depletion of its sole inhibitor CAST. Calpain hyper activation depends on high Calcium concentration in the cytosol. Calpain triggers the production of P35 and the degradation of CAST and PKC. In-Addition, it also triggers the imbalance of calcium homeostasis. All these observations were incorporated into a Stochastic PN models. We gained insight into the mechanism of the AD progression and were able to derive some useful inferences. The first important inference is that under hyper-activation of Calpain, calcium homeostasis dysregulates and CAST starts to degrade gradually. APP processing enzyme (BACE) increases two folds which starts producing *Aβ* that slowly accumulates into plaques in AD brains at early age and lesions appear later. The second important inference is about the most crucial protein Calpain that influences many important proteins of neuronal network such as CAST, PKC, P35, and BACE. Furthermore, these proteins together dysregulate calcium homeostasis. Calpain regulation through CAST plays important role in keeping cells safe from neurodegradation. CAST encounters Calpain at two locations in the cell. Initially at membrane, CAST forms complex with inactive Calpain which is short lived and dissociates as the calcium influx increases. In the cytoplasm, active Calpain-CAST complex is long-standing which keeps the Calpain concentration in control but it also degraded slowly by the action of Calpain. From our study, Calpain and Calpain-CAST complexes have emerged as the potential therapeutic targets for the treatment of neurodegenerative pathologies. The pathway modeling of these networks have predicted that we can introduce delays in the production and accumulation of plaques by targeting the Calpain-CAST complexes. The production of Calpain should be kept in safe levels to avoid its hyper activity. It can be achieved implicitly by enhancing activity of Calpain-CAST complexes. The more durable are the complexes, the lesser would be the accumulation of plaques. Another useful strategy can be the designing of inhibitors against the active Calpain using *in silico* methods and *in vitro* experiments. By considering these effective interventions we can increase the chances of healthy life expectancy and can save many lives and families from the adversity of AD.

## Author contributions

JAs and JAh conceived research idea, designed and performed the experiments, analyzed the data, wrote the paper, prepared figures and/or tables, reviewed drafts of the paper. AA and ZU-H analyzed the data, prepared tables and/or figures, wrote the paper, reviewed drafts of the paper.

### Conflict of interest statement

The authors declare that the research was conducted in the absence of any commercial or financial relationships that could be construed as a potential conflict of interest.

## Footnotes

^1^http://www.alzheimers.net/resources/alzheimers-statistics/
