# Supplementary material for: Analyzing the Behavior of Neuronal Pathways in Alzheimer's Disease Using Petri Net Modeling Approach
Source: Front Neuroinform. 2018 May 23;12:26. doi: 10.3389/fninf.2018.00026 (PMC5974338; doi:10.3389/fninf.2018.00026)
Supplement: Supplementary file 1 [file Data_Sheet_1.pdf]

Supplementary Material

1 PETRI NET ANALYSIS OF ENZYME-SUBSTRATE BRN USING SNOOPY

Petri net (PN) is a powerful formalism which provides a complementary framework for both qualitative and quantitative modeling. It helps in analyzing and simulating the dynamical behavior of large systems in an intuitive way. One exciting feature of PN is its graphical representation which offers construction of BRNs with a biological interpretation and makes it possible to observe and control simulations. The qualitative analysis of BRNs through PN serves as a basis for model validation. Snoopy is a tool to design, animate and simulate graphs in PN. The tool is being used in software based systems as well as in biological systems such as metabolic, signal transduction pathways. It offers different types of PN classes such as qualitative, continuous, hybrid and stochastic PN classes. Snoopy has different modes e.g. editor mode for PN construction, animation mode, simulation mode and model checking mode. A general enzymatic reaction is modeled snoopy software Figure S1a. The model consists of four places: ***Substrate***, ***Enzyme***, Enzyme-substrate Complex (***ESComplex***) and ***Product***. There are also three transitions namely *Association*, *Synthesis* and *Dissociation*. The ***Enzyme*** together with the ***Substrate*** forms the ***ESComplex*** by following the *Association* transition. At the same time, *Dissociation* dissociates the ***ESComplex*** into the ***Enzyme*** and the ***Substrate***. The ***ESComplex*** forms the ***Product*** through *Synthesis* reaction and releases ***Enzyme***.
The simulations launched are shown in Figure S2b. The simulations result accurately demonstrates

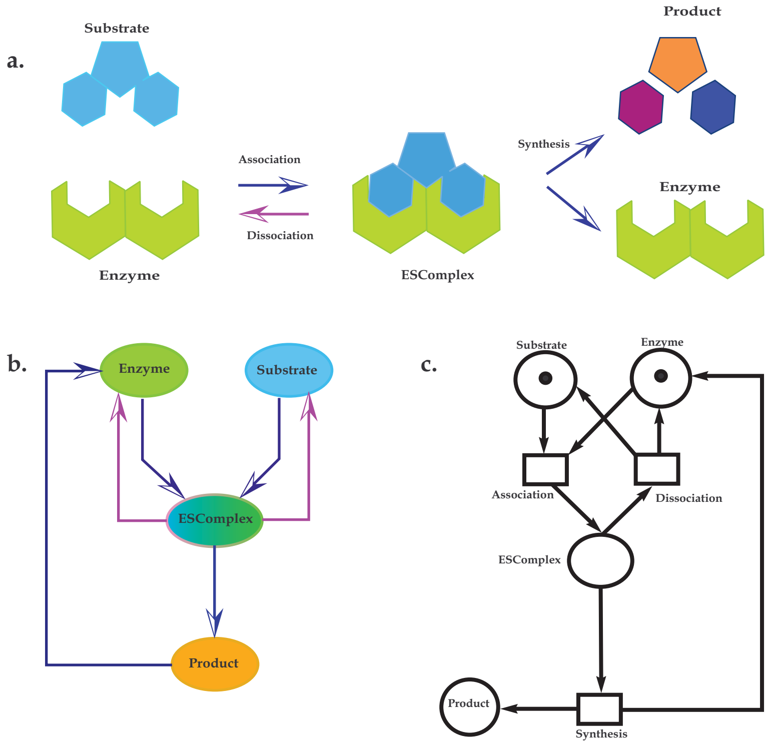

Figure S1: (a) A simple enzymatic reaction consisting of enzyme and substrate which can synthesize product. (b) The BRN of the Enzyme-Substrate reaction. (c) The PN model of Enzyme-Substrate reaction.

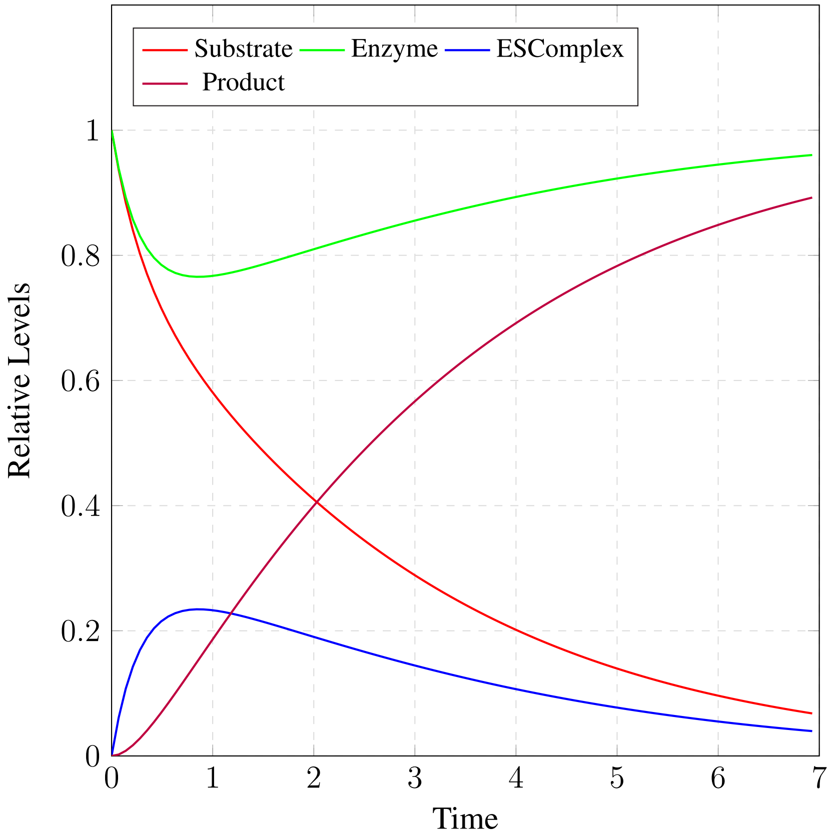

Figure S2: The simulation of the PN model (Figure S1a). The ***Substrate*** is converted into **Product** in the presence of ***Enzyme*** which is recycled and ***ESComplex*** is a short lived complex.

the biological phenomenon of enzymatic reaction that is taking place in living organisms. ***Substrate*** is consumed in the presence of ***Enzyme*** then an intermediate product ***ESComplex*** is formed for a short interval. Eventually, the ***ESComplex*** converts into the ***Product*** and then ***Enzyme*** is recycled.
